# Comparative Chemical Profiling and Antimicrobial/Anticancer Evaluation of Extracts from Farmed versus Wild *Agelas oroides* and *Sarcotragus foetidus* Sponges

**DOI:** 10.3390/md21120612

**Published:** 2023-11-26

**Authors:** Despoina Varamogianni-Mamatsi, Maria João Nunes, Vanda Marques, Thekla I. Anastasiou, Eirini Kagiampaki, Emmanouela Vernadou, Thanos Dailianis, Nicolas Kalogerakis, Luís C. Branco, Cecília M. P. Rodrigues, Rita G. Sobral, Susana P. Gaudêncio, Manolis Mandalakis

**Affiliations:** 1 Hellenic Centre for Marine Research, Institute of Marine Biology, Biotechnology and Aquaculture, 71500 Heraklion Crete, Greece; d.varamogianni@hcmr.gr (D.V.-M.); theanast@hcmr.gr (T.I.A.); e.kagiampaki@hcmr.gr (E.K.); e.vernadou@hcmr.gr (E.V.); thanosd@hcmr.gr (T.D.); 2 School of Chemical and Environmental Engineering, Technical University of Crete, 73100 Chania, Greece; nicolas.kalogerakis@enveng.tuc.gr; 3 Associate Laboratory i4HB, Institute for Health and Bioeconomy, NOVA School of Science and Technology, NOVA University of Lisbon, Campus Caparica, 2819-516 Caparica, Portugal; rgs@fct.unl.pt; 4 UCIBIO—Applied Molecular Biosciences Unit, Chemistry and Life Sciences Departments, NOVA School of Science and Technology, NOVA University of Lisbon, Campus Caparica, 2819-516 Caparica, Portugal; 5 LAQV, REQUIMTE, Associated Laboratory for Green Chemistry, Chemistry Department, NOVA School of Science and Technology, NOVA University of Lisbon, Campus Caparica, 2819-516 Caparica, Portugal; mjm.nunes@fct.unl.pt (M.J.N.); l.branco@fct.unl.pt (L.C.B.); 6 Research Institute for Medicines (iMed.ULisboa), Faculty of Pharmacy, Universidade de Lisboa, Av. Professor Gama Pinto, 1649-003 Lisboa, Portugal; vismsmarques@ff.ulisboa.pt (V.M.); cmprodrigues@ff.ulisboa.pt (C.M.P.R.)

**Keywords:** porifera, demospongiae, marine sponge farming, chemical fingerprinting, marine natural products, secondary metabolites, primary metabolites, MS/MS dereplication, bioactive compounds supply, aquaculture and fish farming

## Abstract

Marine sponges are highly efficient in removing organic pollutants and their cultivation, adjacent to fish farms, is increasingly considered as a strategy for improving seawater quality. Moreover, these invertebrates produce a plethora of bioactive metabolites, which could translate into an extra profit for the aquaculture sector. Here, we investigated the chemical profile and bioactivity of two Mediterranean species (i.e., *Agelas oroides* and *Sarcotragus foetidus*) and we assessed whether cultivated sponges differed substantially from their wild counterparts. Metabolomic analysis of crude sponge extracts revealed species-specific chemical patterns, with *A. oroides* and *S. foetidus* dominated by alkaloids and lipids, respectively. More importantly, farmed and wild explants of each species demonstrated similar chemical fingerprints, with the majority of the metabolites showing modest differences on a sponge mass-normalized basis. Furthermore, farmed sponge extracts presented similar or slightly lower antibacterial activity against methicillin-resistant *Staphylococcus aureus*, compared to the extracts resulting from wild sponges. Anticancer assays against human colorectal carcinoma cells (HCT-116) revealed marginally active extracts from both wild and farmed *S. foetidus* populations. Our study highlights that, besides mitigating organic pollution in fish aquaculture, sponge farming can serve as a valuable resource of biomolecules, with promising potential in pharmaceutical and biomedical applications.

## 1. Introduction

While aquaculture constitutes the fastest growing food production system [1], it can exert pressure on adjacent marine habitats, mainly through the release of organic load and other substances [2]. Among bioremediation candidates to reduce organic pollution, marine sponges have attracted widespread interest [3,4,5,6], due to their innate filter-feeding properties to remove particles (e.g., bacteria [7,8,9,10,11,12], phytoplankton [13,14,15,16]), and dissolved organic [17,18,19]/inorganic substrates [20,21], as well as fish farm wastes [22,23,24] from seawater. Besides their high cleanup capacity, marine sponges also hold great biotechnological potential, and their biomass can be turned into products of high added-value, serving as an additional source of profit for aquaculture-related enterprises. In fact, to date, more than 5000 structurally characterized metabolites have been isolated from marine sponges, contributing to about 30% of all marine natural products [25]. Although a variety of sponges is currently known to produce an overwhelming array of secondary metabolites with pharmaceutical potential [26], the problem of their supply still remains a typical limiting factor for pre-clinical evaluation [27] and further drug development [28].

Over the years, various techniques have been proposed to overcome this bottleneck. Synthesis of bioactive natural products or their related analogues (e.g., via chemical or microbial processes) has always been the preferred method for drug manufacture in pharmaceutical industry. Some of the few successful sponge-derived examples of this strategy are the well-known drugs adenine arabinoside (Ara-A, Vidarabine^®^) and cytosine arabinoside (Ara-C, Cytarabine^®^). Both synthetic products constitute derivatives of sponge nucleosides [29,30], and they have been clinically approved for use as antiviral and antitumor drugs, respectively [31,32,33,34]. However, the development of synthetic strategies for the production of other marine metabolites with greater structural complexity is often challenging and economically unfeasible, even for the purpose of preclinical testing [26,27].

Wild harvesting of sponges that are prolific sources of bioactive compounds has commonly been suggested as a method to supply novel therapeutic agents. However, the typically low naturally occurring concentrations of produced bioactive metabolites, combined with valid concerns for the conservation of sponge diversity in marine ecosystems, are the main reasons to consider this method unsuitable [27,35,36]. This also aligns with the responsible research and innovation aspects that comply with environmental and societal values [37,38]. Nonetheless, a variety of novel marine drug-leads have proceeded to preclinical and clinical trials using materials from wild harvesting. This is the case for avarol, a novel sesquiterpenoid hydroquinone, which is uniquely found in the abundant Mediterranean species *Dysidea avara* [39]. Being one of the most popular sponge-derived bioactive compounds, avarol exhibited strong anti-HIV activity during its preliminary testing [40], but it was later withdrawn from human clinical trials. Moreover, avarol was patented as an anti-psoriasis agent [41], being used in paramedic medicine as one of the ingredients of topical ointments against psoriasis.

Halichondrin B, a metabolite isolated from the sponge *Lissodendoryx* sp., was reported by Hirata and Uemura (1986) [27] as a strong antitumor agent, with a special effect against several melanoma types and leukemia. Although this compound has entered phase I of clinical trials [28], it was produced at very low concentrations by harvested sponges. The cytotoxic metabolite peloruside A, which is isolated from specimens of the sponge *Mycale hentscheli*, is another highly promising antitumor agent, since it operates in a similar way as the anticancer drug Taxol^®^, used to treat ovarian and breast cancers [36]. However, similarly to halichondrin B, wild sponge populations were unable to provide high yields of this compound to proceed in further drug development [42].

Other methods, such as cell lines, primmorphs, and ex situ culture, have extensively been investigated as economically feasible approaches for obtaining sufficient quantities of drug-lead sponge molecules, but they have shown a number of limitations (e.g., high time and resources consumption, poor growth rates) [43,44,45]. By taking into account the plant-like regeneration capability of sponges, and their resilience to overcome physical damage [46], mariculture of these metazoans merges as a promising, cost-effective outlook for the sufficient and sustainable supply of biologically active metabolites [47]. In combination with their filter-feeding characteristics, setting up a sponge farm in proximity to aquaculture operations is becoming highly appealing, since it can promote bioremediation applications through profitability [48].

However, it is of great importance to reassure farmers that the cultivated sponge fragments are able to reproduce the targeted bioactive metabolites of their wild counterparts. In this context, we performed comparative chemical profiling of sponge crude extracts, obtained from wild and farmed populations. The study focused on two widely distributed Mediterranean species, namely *Agelas oroides* and *Sarcotragus foetidus*, which have already been distinguished for their in vitro bioremediation efficiency against various pollutants [16,24] and their production of metabolites with pharmaceutical and biotechnological importance [49,50,51,52]. Chemical characterization of sponge extracts was performed by using high-resolution analytical techniques, such as liquid chromatography coupled with tandem mass spectrometry (LC–MS/MS), targeted to the bioactive metabolites reported in the literature. For this purpose, a comprehensive metabolites/ions/MS fragments library, including all the previously reported compounds for both species, was created. In addition, the sponge extracts were assayed for their biological activities, with a particular focus on their antibacterial and anticancer properties. Through our study, we aspire to demonstrate the bioproduction potential of farmed marine sponges and pave the way for their inclusion as prolific candidates in integrated aquaculture systems.

## 2. Results and Discussion

### 2.1. The Metabolomics Profile of Farmed and Wild Agelas oroides Sponges

The crude extracts obtained from wild and farmed *A. oroides* specimens were analyzed by LC–MS/MS to determine their chemical composition with the aid of a comprehensive metabolites/ions/MS fragments library (Table S1). Three chemical superclasses were identified in the *A. oroides* extracts—alkaloids, indoles, and lipids (Table 1)—with alkaloids being the most predominant constituents in both types of sponge populations (farmed; 97.5%, wild; 98.9%). The majority of the detected alkaloids comprised pyrrole classes, representing 96.2% and 98.3% of the total metabolite content in the farmed and wild *A. oroides* sponges, respectively. Based on skeletal features of the produced pyrrole alkaloids, these were further categorized into three chemical subclasses: (1) linear pyrrole alkaloids, (2) fused cyclic pyrrole alkaloids, and (3) dimeric pyrrole alkaloids [49]. The subclass of terpenoid alkaloids was detected in lower percentages among the farmed and wild sponges (1.3% and 0.6%, respectively). An intriguing class of indole metabolites with unexplored potential in myriad research fields (e.g., chemistry, pharmacology, physiology, and medicine) [53] was also present in the studied extracts, but in significantly low proportions (farmed; 0.03%, wild; 0.02%).

The lipids superclass was present in low abundance and accounted for 2.4% and 1.5% of the total metabolite content in cultured and wild *A. oroides* extracts, respectively. MS^2^ analysis indicated that the extracts with lipid molecules belong to three classes: (1) fatty acyls, (2) glycerolipids, and (3) steroids. The fatty acyls compounds were detected in the following content, (farmed sponges; 2.4%, wild sponges; 1.0%) and to a much lower extent, the classes of glycerolipids and steroids (<0.01%).

The individual components detected in *A. oroides* extracts, along with their chemical and mass spectrometric characteristics are summarized in Table S1. In total, 28 metabolites were identified in wild *A. oroides* extracts, with only three of these compounds being completely absent in the farmed sponge samples (i.e., ageliferin [54], oxysceptrin [55], and trichodermanone C [56]). However, it should be highlighted that these specific compounds were detected in only one replicate of the wild sponges, at significantly low levels (i.e., <0.01%).

The content of each metabolite, expressed by its relative abundance (%) in the tested samples, is presented in Table S2. Based on these results, the linear pyrrole–imidazole alkaloid oroidin, which was identified using electrospray ionization in positive ion mode (ESI+), was the most abundant metabolite found in both farmed and wild *A. oroides* populations (70.3% and 80.4%, respectively). Oroidin constitutes the characteristic metabolite of *A. oroides* sponges [57,58,59]. In fact, it was the first metabolite isolated from this species [60] and possesses broad-spectrum biological activities (e.g., antibacterial, antifouling, antimalarial properties and anti-predatory defenses against reef fish) [49].

Apart from oroidin, abundance differences between farmed and wild sponges were observed in other detected metabolites. Regarding cultivated sponges, the following most abundant metabolites were the closely oroidin-related molecules keramadine [61] (8.0%) and dispacamide B [62] (7.8%). Their respective levels in wild extracts were determined to be as high as 3.6% and 2.9%, respectively. Conversely, the second major metabolite of wild sponges (i.e., 6.8%) was the oroidin hydrolysis product 4,5-dibromopyrrole-2-carboxylic acid [63]. This metabolite was detected at similar abundance levels in the extracts of farmed *A. oroides* specimens (6.6%) and yet was the fourth major metabolite of these extracts. Additionally, small amounts of the linear pyrrole alkaloids dispacamide A [62] and hymenidin [64] (farmed; 1.6–1.0%, wild; 2.1–1.4%, respectively) were determined in both sponge populations.

The differences observed in the ranking of metabolites between wild and cultivated specimens are partially in contrast with the findings of Rodriguez et al. (1994) [65]. In the reported study, it was found that *Acanthella cavernosa* sponges, collected from natural habitats and populations held in controlled aquaculture systems, shared common major constituents, but with different contents in their respective chemical profiles. In our case, oroidin was the common dominant metabolite among studied extracts, but the chemical composition thereafter seemed to be population-specific.

In addition to linear pyrrole alkaloids, which determined the overall metabolite composition of both farmed (97.2%) and wild (95.1%) extracts, compounds belonging to other alkaloid subclasses were also present. Dibromophakellin, an analogue derived through oroidin cyclization/oxidation processes [49,66], previously described from the sponge *Phakellia flabellata* [67], was the most abundant fused cyclic pyrrole alkaloid; however, it was detected at a low percentage (i.e., <1.0%) in both types of extracts. This was followed by its relative congeners longamide B methyl ester [68], longamide B [69], monobromoisophakellin [70], and 3-debromohanishin [71]. Overall, the percentage of fused cyclic pyrrole alkaloids reached up to 0.7% and 0.9% for cultured and wild *A. oroides* specimens.

Dimeric pyrrole alkaloids, formed by oxidation, cyclization, and dimerization reactions of simple monomers (i.e., oroidin, clathrodin, and hymenidin) [72] was the least abundant alkaloids group within the extracts (farmed; 0.4% and wild; 0.2%). Nakamuric acid [73] and debromosceptrin acetate [74] represented the majority of such metabolites, while bromoageliferin [54] and sceptrin [75] were present in very low percentages (<0.01%).

Terpenoid alkaloids, which involve a nitrogen-based functional group (in the form of an amine or ammonia, etc.) attached to preformed terpenoid moieties [49,76], were also present at low levels in *A. oroides* extracts (up to 1.1% for farmed and 0.5% for wild sponges). This was the case for metabolites belonging to the families of agelasines (e.g., agelasine [77], agelasine A [78] and E [79]), as well as agelasidines (e.g., agelasidine A [80]).

The lipid content of both farmed and wild *A. oroides* was mainly characterized by fatty acyls, which were dominated by the compound 10-methyl-9(*Z*)-octadecenoic acid [81], accounting for 2.4% and 1.0% of the total metabolite composition in the cultured and wild sponge extracts, respectively. Interestingly, this unsaturated fatty acid has previously only been recorded in extracts of the marine fungus *Microsphaeropsis olivacea*, which was isolated by Yu et al. (1996) from a sponge collected in Florida. Along with Tasdemir et al. (2007) [82], who previously reported the presence of the isomer 11-methyloctadecanoic acid in *A. oroides*, we are the first to discover the existence of 10-methyl-9(*Z*)-octadecenoic acid in this sponge species. Its glyceride [81] was also detected, but at trace concentrations in both types of extracts (i.e., <0.003%). Detected steroids were mainly members of the ecdysteroids class, such as 20-hydroxyecdysone-22-acetate [83], β-ecdysterone [69] and ponasterone A [83], which collectively accounted for <0.01% of the sponge extracts.

5,6-dihydroxyindole was the only representative of the indole family. However, it was present in very low concentrations for both farmed (0.03%) and wild (0.02%) sponge extracts. This metabolite is an intermediate of the melanin biosynthetic pathway, which has previously demonstrated antibacterial activity against Gram-negative (e.g., *Escherichia coli*) and Gram-positive bacterial pathogens (e.g., *Staphylococcus aureus*), as well as antifungal activity [84].

**Figure 1:**
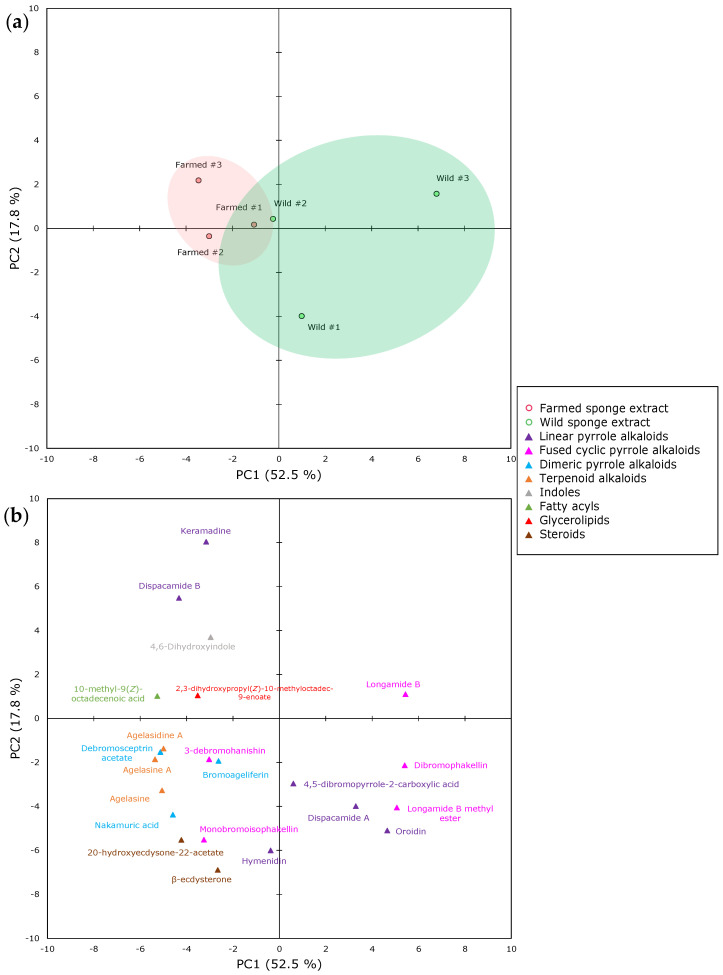
Principal component analysis (**a**) scores plot and (**b**) loadings plot of crude *A. oroides* extracts belonging to wild and farmed sponges. Color circles represent the origin of the analyzed sponge extract (i.e., green for wild or red for farmed) and triangles represent the different chemical subclasses of the identified metabolites, according to the legend.

The metabolic profiling of the crude sponge extracts was further investigated by an unsupervised principal component analysis (PCA), to evaluate the similarities and differences between the extracts of the two populations and access clustering trends, as well as identifying outliers. The results obtained from the PCA (Figure 1) indicated the high spatial distribution of the extracts produced by wild *A. oroides* fragments, which are highlighted in green. Extracts derived from farmed specimens (highlighted in red) are closely clustered. Based on these findings, it can be assumed that *A. oroides* sponges are likely to provide extracts with a similar chemical profile when subjected to farm conditions, whereas the composition of the wild individuals can be more diverse. This result presumably reflects the more homogenous environment of the farm conditions, in contrast to the complexity of a natural habitat, where the biodiversity assemblages and interactions between their organisms at micro and macro levels are more complex and intricate, thus inducing diversification to the sponges’ chemical phenotype. However, according to the PCA scores plot (Figure 1a), the extracts derived from the farmed sponges are closely similar to one of the wild specimens, supporting a consistency of the core chemical profile of *A. oroides*, disregarding the prevalent drivers in the natural versus aquaculture environments.

In a similar study, Page et al. (2005) [85] investigated the biosynthesis of metabolites from sponges *Mycale hentscheli*, collected from aquaculture and natural habitats in New Zealand. Their results showed that the levels of the cytotoxic compounds mycalamide A, pateamine, and peloruside A varied not only within wild specimens, but also among farmed explants. Consequently, differences in the chemical profile were evident at an individual-specific level, rather than among different populations. Similar findings were reported for the bioactive metabolite amphitoxin, produced by cultured and natural species of the Indonesian reef-dwelling sponge *Callyspongia biru* [86].

Studies have shown that the variability of secondary metabolite production in sponges can be pronounced at the intraspecific level, among populations or even among individuals [42,87,88]. A variety of biological traits (e.g., sponge shape [89] and size [90]) or environmental factors (e.g., response to predation [91], pollutants [92], light [93,94], and temperature [42]) can induce these marine organisms to modify their levels of secondary metabolites. Considering the fluctuation of the aforementioned parameters in natural habitats, compared to the less complex artificial environment of a fish farm, a higher diversity of secondary metabolites is expected for wild versus farmed sponge specimens.

Overall, the first two principal components (PC1 and PC2) explained 52.5% and 17.8% of the total variance present in the dataset (Figure 1). Interestingly, the loadings plot (Figure 1b) shows that the metabolites highly enriched in carbon content (i.e., C_22_–C_29_), such as dimeric pyrrole and terpenoid alkaloids, as well as steroids, are grouped together in the bottom left quadrant of the panel. As was expected, the linear pyrrole alkaloids oroidin and 4,5-dibromoprrole-2-carboxylic acid, which constitute the major constituents of the wild specimens, are scattered closely to the wild extracts. The same stands for the linear pyrrole alkaloids keramadine and dispacamide B with respect to farmed sponges.

**Figure 2:**
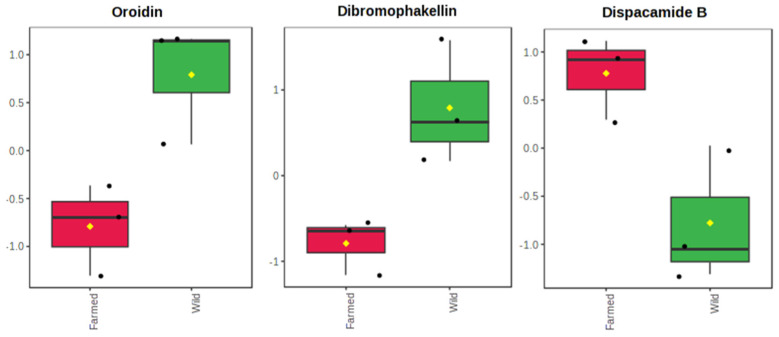
Boxplot of relative abundances for metabolites oroidin, dibromophakellin, and dispacamide B, which presented significant abundance differences (*t*-test, *p* < 0.05) between the extracts content of farmed (red) and wild (green) *A. oroides* sponges. Data were normalized to the total spectral area and presented in the auto-scale mode of the MetaboAnalyst 5.0 software. Boxes range from the 25% to 75% percentile; whiskers indicate the 5% and 95% percentiles; black dots show the individual data points; the horizontal line and the yellow diamond within each box indicate the median and mean value, respectively.

However, a series of *t*-tests (Figure 2) revealed that significant differences in the metabolite content were observed only for the bromopyrrole alkaloids oroidin (*p* = 0.025) and dibromophakellin (*p* = 0.025), which were richer in wild *A. oroides* sponges. Furthermore, compounds of the same class, including ageliferin and oxysceptrin, were completely absent in farmed *A. oroides* sponges. Previous studies have highlighted the role of oroidin-like brominated pyrrole alkaloids as chemical defenders against fish predation for the genus *Agelas* [63,95] and enhanced bromine content with increased feeding deterrent potency [96]. Given that sponges occurring in natural habitats are prone to face increased antagonistic interactions compared to their farmed counterparts, a higher content of bromopyrrole alkaloids can thus be expected in wild specimens.

Interestingly, farmed *A. oroides* sponges exhibited a significantly higher content in the pyrrole alkaloid dispacamide B (*p* = 0.031), which was also included in the top three abundant metabolites of the respective extracts (Figure 2). However, the ecological role of this alkaloid has not yet been well established. It is likely to be related to other biological functions of this species, such as growth or reproduction, but a lack of evidence means its higher biosynthesis in the farmed population cannot be explained. Nevertheless, oroidin, dibromophakellin, and dispacamide B could serve as chemotaxonomic markers for distinguishing farmed from wild *A. oroides* specimens, but further investigation is required to support this statement. Overall, the distribution of the various metabolite classes between the two sponge populations remained constant, but the composition of the three above mentioned compounds varied according to the population.

**Figure 3:**
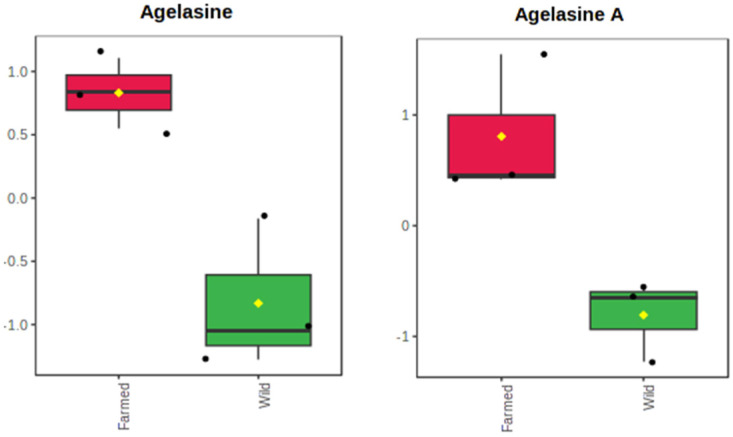
Boxplot of weight-normalized intensities for metabolites agelasine and agelasine A, which presented significant differences in their production (*t*-test, *p* < 0.05) between the extracts of farmed (red) and wild (green) *A. oroides* sponges. Data are presented in the auto-scale mode of the MetaboAnalyst 5.0 software. Boxes range from the 25% to 75% percentile; whiskers indicate the 5% and 95% percentiles; black dots show the individual data points; the horizontal line and the yellow diamond within each box indicate the median and mean value, respectively.

Apart from assessing differences in the metabolite content, we further aimed to compare production levels of the respective bioactive compounds in the two sponge populations. For the quantification of the detected metabolites, we introduced the term of the weight-normalized intensity, that considers the dry weight of each sponge fragment subjected to extraction (expressed as intensity units per gram of sponge dry weight). The calculated values are presented in Table S3. Out of the 25 metabolites shared between farmed and wild sponges, only two showed significant variation in their production levels. These were the terpenoid alkaloids agelasine (*t*-test, *p* = 0.012) and agelasine A (*t*-test, *p* = 0.019), which were increasingly produced by farmed explants (Figure 3).

A handful of studies have similarly indicated that farming can promote the production of specific bioactive metabolites in sponges. By following a 9-month strategy of harvesting explants from cultures of the New Zealand demosponges *Latrunculia wellingtonensis* and *Polymastia croceus*, Duckworth et al. (2003) [47] measured similar or higher bioactivity in farmed specimens than that exhibited by natural populations. However, it should be noted that the overall metabolite production was estimated in terms of a murine leukemia bioassay, and not by using metabolomics approaches.

Furthermore, an array of ex situ trials revealed that farmed sponge explants are greater producers than their wild counterparts with respect to specific biomolecules. This was the case for the Mediterranean sponge *Aplysina aerophoba*, and its brominated isoxazoline alkaloids aplysinamisin-1, aerophobin-2, and isofistularin-3, as well as the biotransformation product aeroplysinin-1, when explants of the specific species were kept for 9 months in controlled aquarium systems [97]. Similar to the previous work, Duckworth et al. (2003) reported higher levels of the antitumor compound stevensine in cultures of *Axinella corrugata*, by following a multicomponent diet [98]. Contrarily, Munro et al. (1999) [28] found that the overall halichondrin content of the cultured *Lissodendoryx* sponge was not as high as that of the wild specimens, reaching the opposite conclusion. No effect of farming on metabolite production was indicated by Carballo et al. (2010) [27] and Ternon et al. (2017) [99], who conducted sponge mariculture of the species *Mycale cecilia* and *Crambe crambe*, respectively.

### 2.2. The Metabolomics Profile of Farmed and Wild Sarcotragus foetidus Sponges

The LC–MS/MS analysis of *S. foetidus* crude extracts revealed that, in total, 31 metabolites were shared between farmed and wild sponges, with all the compounds being present in at least one biological replicate of each population (Tables S4 and S5). Detection was supported by building a comprehensive metabolites/ions/MS fragments library (Table S4). The detected *S. foetidus* metabolites were classified into five superclasses, namely benzenoids, dipeptides, indoles, lipids, and polyketides (Table 2). This species was revealed to be an efficient lipid producer, by presenting extracts with an average lipid content as high as 95.5% and 94.3% among wild and farmed specimens, respectively. The detected lipids classes were: (1) fatty acyls, (2) glycerolipids, (3) prenol lipids, and (4) steroids. This slightly higher predominance of lipids observed in the extracts of farmed sponges was evaluated as statistically significant (*t*-test, *p* = 0.002), and is attributed to the higher levels of prenol lipids (*t*-test, *p* = 0.011) in the respective extracts when compared to their wild counterparts. However, this specific class held only a small share in the overall chemical composition of both sponge populations (farmed; 3.1% and wild; 1.5%). Lipids were mainly dominated by the fatty acyls class, which accounted for more than 55% of the overall metabolite abundance, followed by steroids, averaging at approximately 28.7% and 30.1% within farmed and wild sponges, respectively. However, this steroid dominance in wild individuals was regarded as marginally significant (*t*-test, *p* = 0.037). Glycerolipids were present at similar abundance levels within the sponge populations, collectively accounting for 7.4% and 7.2% of the farmed and wild sponge extracts.

Benzenoids were the second major compound superclass, and, yet, were only detected at small percentages in both types of extracts (i.e., <4.2%). It comprised four classes, (1) anthracenes, (2) benzene and substituted derivatives, (3) benzopyrans, and (4) phenols. Interestingly, benzenoid moieties were more pronounced in wild specimens (*t*-test, *p* = 0.007), due to their higher content in benzenes and substituted derivatives (*t*-test, *p* = 0.016). Moreover, metabolites belonging to the class of benzopyrans were identified at similarly low levels within the farmed and wild sponge populations (i.e., 0.4% and 0.5%, respectively). Furthermore, other compound classes contributed to the benzenoid profile of farmed and wild *S. foetidus* extracts, such as anthracenes and phenols, but these were present as trace elements (i.e., <0.01%).

The chemical profile of *S. foetidus* exhibited superclasses of polyketides and indoles that were found in all extracts of this keratose demosponge. The respective contents were similar for sponges of farmed and wild populations. More specifically, polyketides accounted for 0.8% of both farmed and wild sponge extracts. Indoles were present at percentages lower than 0.1% in both *S. foetidus* populations. Additionally, dipeptides were the least abundant metabolite superclass among the sponges’ populations (i.e., <0.02%).

The distribution of metabolites within the extracts of *S. foetidus* was determined using the MS^2^ peak intensity data, and the calculated abundance values are presented in Table S5. Fatty acyls of farmed and wild sponge extracts were entirely represented by the primary metabolite *N*-hexadecanoyl-*L*-homoserine lactone [100], which has been previously detected in wild specimens of this species through in situ chemical extraction [101] and its existence has been associated with the quorum-sensing activities of its host microbiome [102]. The steroid 24-methylcholesta-5,7,22-trien-3β-ol (i.e., ergosterol) [103] was the second most abundant metabolite, and it accounted for the majority of steroids detected in *S. foetidus* extracts. Its content was determined to be as high as 28.6% and 30.0% in sponges derived from farmed and wild sponges, respectively.

Monovaccenin [104] and 1-*O*-(2,3,4,5-tetrahydroxycyclopentyl)-3-*O*-(10-methylhexadecyl)glycerol (1-*O*-(2,3,4,5-4OHCyp-3-*O*-10-MeHG)) [105] were the major compounds of the glycerolipids family, and their respective average percentages were 4.6% and 2.5% within the analyzed extracts. However, the prenol lipid 4-hydroxy-3-tetraprenylbenzoic acid [103] was detected at similar levels as 1-*O*-(2,3,4,5-4OHCyp-3-*O*-10-MeHG), but only in farmed specimens (i.e., 2.6%). Wild extracts exhibited approximately two times lower percentages of this compound (i.e., 1.3%). The opposite trend was observed for the benzenoid molecule toluate (i.e., 2.1% and 1.5% in wild and farmed sponges, respectively), while the second major benzenoid 8-*O*-4’-dehydrodiferulic acid [106] accounted for 1.3% and 1.6% of the total metabolite content of farmed and wild sponge populations.

Other compounds belonging to benzenoids (i.e., 3-phenylpropane-1,2-diol [106], 3-isochromanone [107], and 7-hydroxy-2-(2-hydroxypropyl)-5-methylchromone [108]), indoles (i.e., indole-3-methylethanoate [109]), lipids (i.e., 1-*O*-(2,3,4,5-tetrahydroxycyclopentyl)-3-*O*-(11-hexadecenyl)glycerol, 1-*O*-(2,3,4,5)-4OHCyp-3-*O*-HG [105], and 1,4-dihydroxy-2-tetraprenylbenzoic acid [103]) and polyketides (i.e., 3,8-dihydroxy-6-methoxy-8-methylxanthone [110], chrysophanol [111], and griseofulvin [112]) exhibited minor abundances (i.e., 0.1-0.4%) in relation to the total metabolite content of *S. foetidus*. Anthracenes, represented by emodin [113] and endocrocin [114], and dipeptides comprised 3-nitropropionic acid [115], along with some benzene derivatives (i.e., 3,4-dimethoxybenzoic acid [116], 4-hydroxyphenylacetic acid [117], tyrosol [118]), prenol lipids (i.e., 7*E*,12*E*,20*Z*-variabilin [119] and (+)-12,15-dihydroxycurcuphenol [120]), steroids (i.e., 24-methylcholest-7-en-3β-ol, 24-methylcholesta-5,7-dien-3β-ol, 24-methylcholesta-7,22-dien-3β-ol, cholest-7-en-3β-yl acetate, and cholesta-5,7-dien-3β-ol [121]), and polyketides (i.e., dechlorogriseofulvin [112], and norlichexanthone [122]) were detected as trace amounts within the tested extracts (i.e., <0.1%).

**Figure 4:**
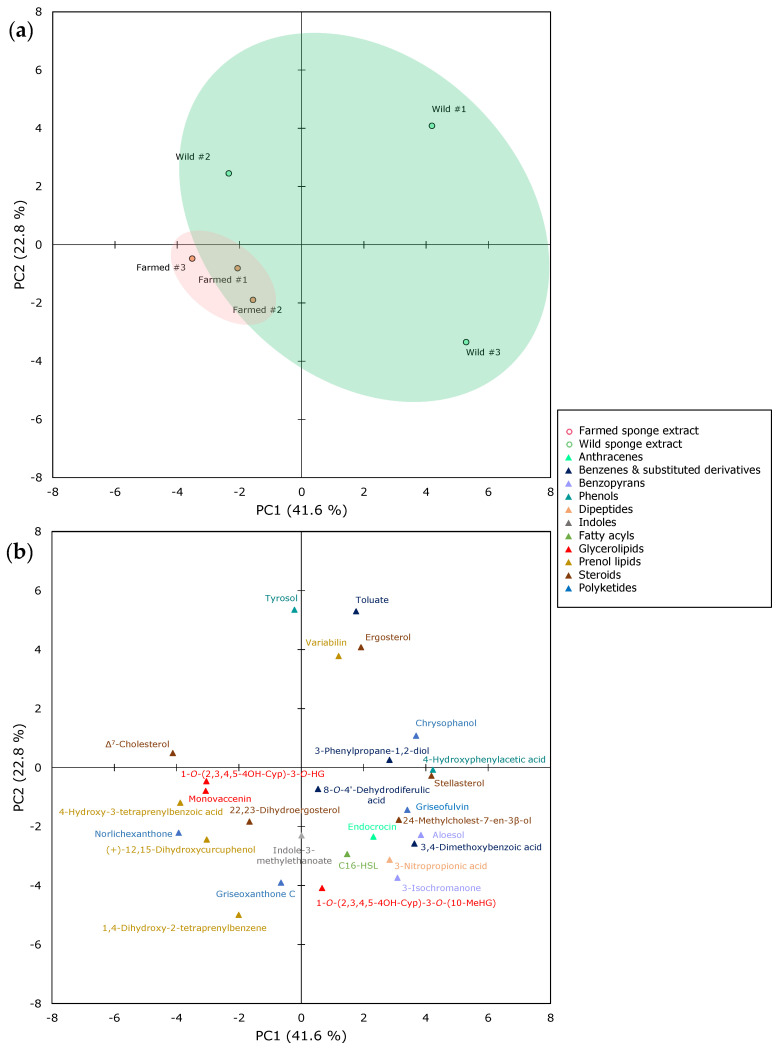
Principal component analysis (**a**) scores plot and (**b**) loadings plot of crude *S. foetidus* extracts belonging to wild and farmed sponges. Color circles represent the origin of the analyzed sponge extract (i.e., green for wild or red for farmed) and triangles represent the different chemical classes of the identified metabolites, according to the legend: 1-*O*-(2,3,4,5)-4OHCyp-3-*O*-HG, 1-*O*-(2,3,4,5-tetrahydroxycyclopentyl)-3-*O*-(11-hexadecenyl)glycerol, 1-*O*-(2,3,4,5-4OHCyp-3-*O*-10-MeHG, 1-*O*-(2,3,4,5-tetrahydroxycyclopentyl)-3-*O*-(10-methylhexadecyl)glycerol); aloesol, 7-hydroxy-2-(2-hydroxypropyl)-5-methylchromone; C16-HSL, *N*-hexadecanoyl-*L*-homoserine lactone; Griseoxanthone C, 3,8-dihydroxy-6-methoxy-8-methylxanthone; Δ^7^-Cholesterol, Cholesta-5,7-dien-3β-ol; 22,23-Dihydroergosterol, 24-methylcholesta-5,7-dien-3β-ol; stellasterol, 24-methylcholesta-7,22-dien-3β-ol; ergosterol, 24-methylcholesta-5,7,22-trien-3β-ol.

The compositional differences between the extracts derived from farmed and wild *S. foetidus* sponges were further evaluated by PCA analysis. Similar to the case of *A. oroides*, data points representing the chemical profiles of wild sponges showed a broader distribution, while a clear clustering was observed for the samples obtained from farmed explants (Figure 4a). The first two principal components (PC1 and PC2) explained 41.6% and 22.8% of the total variance present in the dataset.

**Figure 5:**
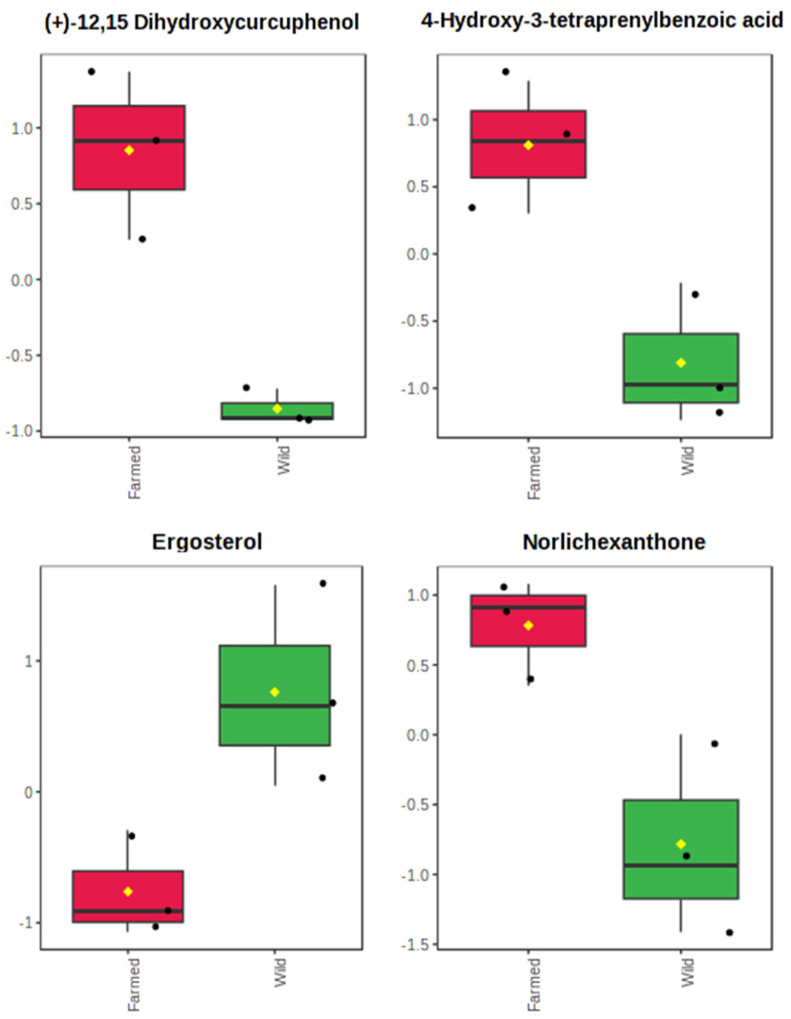
Boxplot of relative abundances for metabolites (+)-12,15 dihydroxycurcuphenol, 4-hydroxy-3-tetraprenylbenzoic acid, ergosterol, and norlichexanthone, which presented significant abundance differences (*t*-test, *p* < 0.05) between the extracts content of farmed (red) and wild (green) *S. foetidus* sponges. Data were normalized to the total spectral area and presented in the auto-scale mode of the MetaboAnalyst 5.0 software. Boxes range from the 25% to 75% percentile; whiskers indicate the 5% and 95% percentiles; black dots show the individual data points; the horizontal line and the yellow diamond within each box indicate the median and mean value, respectively.

Several members of lipids superclass, belonging to the classes of glycerolipids (i.e., 1-*O*-(2,3,4,5)-4OHCyp-3-*O*-HG and monovaccenin), prenol lipids (i.e., (+)-12,15-dihydroxycurcuphenol, 1,4-dihydroxy-2-tetraprenylbenzoic acid, and 4-hydroxy-3-tetraprenylbenzoic acid), and steroids (i.e., Δ^7^-cholesterol and 22,23-dihydroergosterol), together with the majority of detected polyketides (i.e., griseoxanthone C and norlichexanthone), are visually clustered in the bottom left side of the PCA loadings plot (Figure 4b), corresponding to the extracts derived from farmed *S. foetidus* sponges in the scores plot (Figure 4a). This supports the notion that these specific compounds might be the key elements accounting for the variation presented in the farmed and wild sponges’ chemical profiles. To shed light onto this pattern, we tested the differences in the % abundance of each metabolite between farmed and wild sponges. The results showed that, indeed, some of the abovementioned compounds were significantly higher in abundance in farmed *S. foetidus* explants, namely the prenol lipids (+)-12,15-dihydroxycurcuphenol (*p* = 0.006) and 4-hydroxy-3-tetraprenylbenzoic acid (*p* = 0.018) and the polyketide norlichexanthone (*p* = 0.029) (Figure 5). Furthermore, wild *S. foetidus* specimens were enriched in ergosterol (*p* = 0.039), which was illustrated in the upper right side of PCA biplot, close to one of the wild samples.

All the lipids that varied in abundance between farmed and wild *S. foetidus* populations have been associated with sponge defense mechanisms. More specifically, (+)-12,15-dihydroxycurcuphenol constitutes an intermediate metabolite in the synthesis of abscisic acid, which is assumed to be produced by sponges as a response to heat stress [120]. Additionally, the compound 4-hydroxy-3-tetraprenylbenzoic acid has been shown to possess strong pungent activity [123], potentially serving as a defensive compound by the sponges to deter predators. Norlichexanthone is a marine-derived fungi metabolite [122], which has been previously reported in the fungal extracts of the *Sarcotragus muscarum* symbiont *Arthrinium* sp. [106]. Ahluwalia et al. (2015) [124] included this metabolite in high oxidation state compounds, which have been previously reported to exhibit great antimicrobial activity, while boosting symbionts’ competitive efficiency and host resistance to other pathogens [125]. However, all these defensive lipid chemicals were found to be more abundant in farmed populations of *S. foetidus*, which is quite contradictory, given the lesser environmental pressures they face, compared to their wild counterparts. However, it should be mentioned that extracts provided by farmed *S. foetidus* specimens are less rich in ergosterol. This specific compound is the prime sterol in plasma membranes of fungal cells, and like many steroids, has been characterized as a bioactive compound, due to its antibacterial and anti-inflammatory activities [126]. Higher levels of this fungi steroid in wild sponge extracts indicate a higher pronounced presence of symbionts in the respective specimens and their potential involvement in the sponge-associated defense mechanisms. Taking this into account, we can perceive why the metabolism of farmed *S. foetidus* sponges involved alternative defense pathways, enriched with other minor metabolites, such as prenol lipids.

**Figure 6:**
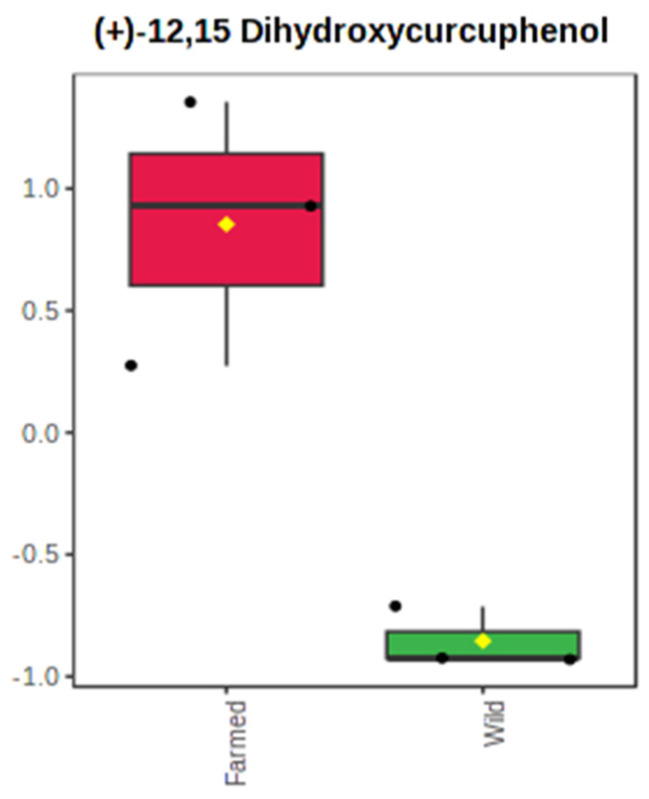
Boxplot of weight-normalized intensities for the metabolite (+)-12,15 dihydroxycurcuphenol, which presented significant differences in its production (*t*-test, *p* < 0.05) between the extracts of farmed (red) and wild (green) *S. foetidus* sponges. Data are presented in the auto-scale mode of the MetaboAnalyst 5.0 software. Boxes range from the 25% to 75% percentile; whiskers indicate the 5% and 95% percentiles; black dots show the individual data points; the horizontal line and the yellow diamond within each box indicate the median and mean value, respectively.

In terms of production levels, the weight-normalized intensity values calculated for each metabolite of *S. foetidus* sponges are presented in Table S6. Only the prenol lipid (+)-12,15-dihydroxycurcuphenol was found to vary significantly between farmed and wild *S. foetidus* sponges (*p* = 0.006) (Figure 6). In agreement with the previously reported one-way ANOVA results (i.e., assessing differences in % metabolite abundance among extracts), the production of (+)-12,15-dihydroxycurcuphenol was more intensive in farmed specimens. Consequently, rearing in proximity to a fish farm facility may affect the production efficiency of specific bioactive compounds in *S. foetidus* individuals, but this is an exception to a broader uniformity in metabolite production levels.

### 2.3. Evaluation of the Sponges’ Biotechnological Potential

In addition to comparing the chemical profiles of cultivated and wild sponges, we further examined the biological activities of *A. oroides* and *S. foetidus* extracts to evaluate whether farming can affect those properties. Antimicrobial assays were performed against two human pathogens: methicillin-resistant *Staphylococcus aureus* (MRSA, COL), representing Gram-positive bacteria, and *Escherichia coli* (ATCC 25922), representing Gram-negative bacteria. In addition, the anticancer activities and general toxicities of the extracts were assessed against the human colorectal carcinoma cell line HCT-116 (ECACC 91091005).

#### 2.3.1. Antimicrobial Activity Evaluation

To assess the antibacterial potential of *A. oroides* and *S. foetidus* marine sponges and compare the bioactivities of wild versus farmed populations, a total of 12 extracts were tested against two strains of Gram-positive and -negative bacteria, representative of clinically relevant human pathogens, as mentioned above: *Staphylococcus aureus* (MRSA strain COL) and *Escherichia coli* (strain ATCC 25922). The studied samples consisted of the crude extracts of three explants collected from both farmed and wild populations of targeted sponge species *A. oroides* and *S. foetidus*, analyzed in triplicate, using the microdilution assay to determine the minimum inhibitory concentration (MIC) (Table 3). In total, eight crude sponge extracts revealed antibacterial properties, with all showing marginal potency. More specifically, seven extracts were found to be active against the *S. aureus* strain, while only one was proven to be effective against *E. coli*.

Regarding the species *A. oroides*, one out of the three wild specimens (33%) provided extracts with inhibitory activities against the Gram-positive methicillin-resistant bacterium *S. aureus*, while the same stood for two out of the three studied farmed samples (67%). However, both types of *A. oroides* extracts (i.e., farmed vs. wild) exhibited activity against MRSA, albeit only for the highest tested concentration (i.e., 250 μg/mL).

Interestingly, one farmed *A. oroides* extract (i.e., #3), besides revealing *S. aureus* growth inhibition, also showed activity against *E. coli* (MIC; 250 μg/mL). None of the wild sponge extracts belonging to *A. oroides* demonstrated inhibitory effects against *E. coli*. The same results were observed for farmed *A. oroides* extracts, expect for replicate #3, which targeted both Gram-positive and -negative bacteria, suggesting a mechanism not related to the cell wall. Indeed, among all the tested biological samples, the farmed *A. oroides* replicate #3 extract contained the highest levels of the alkaloids dispacamide B, keramadine, and agelasidine A, as well as the indole compound 4,6-dihydroxyindole and the glycerolipid 2,3-dihydroxypropyl(*Z*)-10-methyloctadec-9-enoate (Table S2). Of these metabolites, keramadine and agelasidine A have been previously reported to display partial growth inhibitory effects against *E. coli* strains, with MIC values in the range of 32–100 μg/mL [127,128], while 4,6-dihydroxyindole was found to have strong antibacterial activity against both Gram-positive and -negative bacteria, including *E. coli* [84]. These compounds might be the key metabolites equipping *A. oroides* sponges with broad-spectrum antibacterial properties, by acting either individually or synergistically in the respective extracts.

In contrast, extracts of wild *S. foetidus* sponges had a stronger effect on *S. aureus* growth, with one of these being active at the highest tested concentration and the rest of the samples reaching lower MIC values of 125 µg/mL. However, it is worth noticing that this value represented the highest antimicrobial activity exhibited by all the studied extracts. Concerning the cultivated *S. foetidus* fragments, only one out of three (33%) generated extracts that marginally inhibited the growth of the Gram-positive bacterium (MIC; 250 µg/mL), but did not reach as low values as their wild counterparts. On the other hand, all the *S. foetidus* extracts did not show any effect on *E. coli* growth.

Based on these observations, we can assume a higher presence of anti-*S. aureus* compounds in the extracts of wild *S. foetidus* populations, which target the cell wall structure, because only Gram-positive bacteria are inhibited. More specifically, wild extracts #1 and #2, which stood out for their inhibitory properties, demonstrated the highest contents of the natural products toluate (i.e., >2.0%) and ergosterol (i.e., >29.9%) among all the tested samples. Toluate, or *p*-toluic acid, is a primary metabolite involved in the natural degradation of *p*-xylene [129] and can be found in a myriad of organisms [130,131], including the sponge *S. foetidus* [101]. To the best of our knowledge, there are no reports examining its antimicrobial effects, except for those focused on its congener, benzoic acid. This compound is a food preservative, and its MIC values against various *S. aureus* strains have been demonstrated in several previous studies [132,133]. On the other hand, ergosterol, a derivative of cholesterol, plays an important role in the function of eukaryotic cell membranes by maintaining their permeability. However, it is absent in the cytoplasmic membrane of bacteria [134]. Tintino et al. (2017) reported that ergosterol causes detrimental effects on bacterial cells [135]. This could be a possible explanation for the enhanced antimicrobial activity observed in the majority of crude extracts collected from wild *S. foetidus* specimens. However, there are no data in the literature describing ergosterol-driven effects on bacteria.

Although the reported MIC values of the present study are relatively high, it should be stressed that they concern the activity of crude sponge extracts, which have not been priorly subjected to any fractionation. In agreement with the results obtained from Govinden-Soulange et al. (2014) [136] for crude and fractionated extracts of the Mauritian sponges *Biemna tubulosa* and *Stylissa* sp., MIC values of our crude samples were rarely found to be higher than 100 μg/mL. This is attributed to the chemical complexity of the specific extracts, which constitute mixtures of both active and non-active compounds.

#### 2.3.2. Anticancer Activity Evaluation

**Figure 7:**
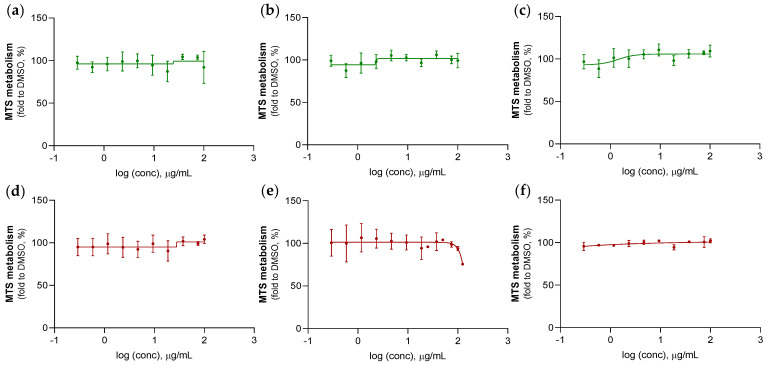
HCT-116 cells viability determined by the MTS assay after a 72-h treatment with extracts derived from wild (**a**–**c**) and farmed (**d**–**f**) *A. oroides* specimens at different concentrations (presented in a logarithmic scale). All data points are expressed as mean ± standard error of the mean from at least three independent experiments. DMSO was used as vehicle control, and 10 µM 5-Fu as positive control.

**Figure 8:**
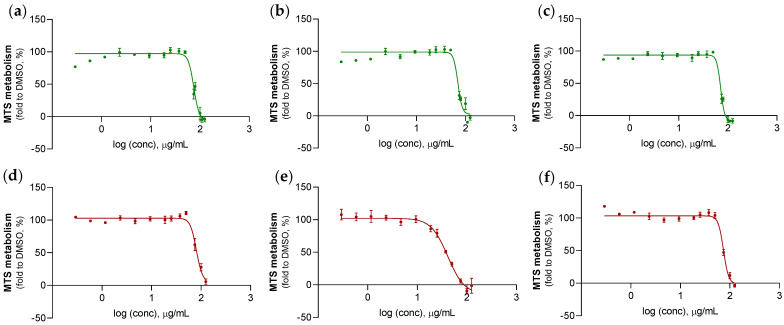
HCT-116 cells viability determined by the MTS assay after a 72-h treatment with extracts derived from wild (**a**–**c**) and farmed (**d**–**f**) *S. foetidus* specimens at different concentrations (presented in a logarithmic scale). All data points are expressed as mean ± standard error of the mean from at least three independent experiments. DMSO was used as vehicle control, and 10 µM 5-Fu as positive control.

The anticancer properties of crude sponge extracts, derived from farmed and wild populations of *A. oroides* and *S. foetidus* species, were investigated against the human colorectal carcinoma cell line HCT-116, by employing the MTS assay. Figure 7 and Figure 8 illustrate the variation of MTS cell metabolism as a function of the applied extract concentration, with respect to *A. oroides* and *S. foetidus* sponges. The cytotoxicity effects were evaluated according to half-maximal (50%) inhibitory concentration (IC_50_) values, which were obtained from the curve slopes of the abovementioned graphs and are presented in Table 4. As shown in Figure 7, none of the extracts belonging to wild and farmed *A. oroides* explants exerted anticancer activity against HTC-116 cells, except for one extract of a farmed specimen (#3, Figure 7f) that showed approximately 25% inhibition at the highest test concentration (i.e., 125 μg/mL). However, this inhibition rate was considered to be relatively low, hence, the IC_50_ values were not determined.

All *S. foetidus* sponges provided extracts with moderate anticancer activity, whether they derived from wild or farmed populations. More specifically, wild explants exhibited IC_50_ values in the range of 70.8–71.5 μg/mL, while their cultivated counterparts showed a more profound variation, with values starting from 80.5 μg/mL and reaching 41.2 μg/mL. However, the average cytotoxicity against HTC-116 cells, which was derived from the IC_50_ values of the three tested sponge extracts, was evaluated as statistically similar between the two populations (*t*-test, *p* = 0.7). This was found to be as high as 70.9 ± 0.5 μg/mL for wild sponges and 65.3 ± 21.1 μg/mL for the farmed ones.

Based on these observations, it can be assumed that *S. foetidus* extracts contain metabolites that are more active against HCT-116 cells than those detected in *A. oroides*. However, previous studies have indicated the cytotoxicity of *Agelas* species against various carcinoma cells, but the associated results concern the activity of isolated metabolites, like oroidin [137] and sceptrin derivatives [138], and not the whole sponge extract. Although these metabolites were present in our tested *Agelas* extracts, it is likely that the levels at which they were produced, remained relatively low to exhibit bioactivity, or other components might counteract their inhibitory properties. Nevertheless, the highest percentage of HCT-116 cell inhibition, observed for one of our farmed *A. oroides* specimens, was within the range reported by Ang et al. (2023) for various crude extracts of *Agelas* species against the HCT-116 carcinoma cell line [139]. However, it should be mentioned that the latter study revealed the lowest percent of cell viability (i.e., 75.8%) at 30 μg/mL, which was four times lower than our “active” concentration (i.e., 125 μg/mL) for crude *Agelas* extracts.

Regarding *S. foetidus*, it can be observed that all study extracts, either derived from wild or farmed specimens, showed 50% inhibition against HCT-116 cells. However, according to the American National Cancer Institute guidelines (NCI), crude extracts achieving 50% anti-proliferative activity are regarded as cytotoxic at less than 30 μg/mL after a 72-h exposure [140]. Based on this, we can consider that only one extract, which belongs to a farmed *S. foetidus* specimen (i.e., #2), is marginally cytotoxic, given its mean IC_50_ value of 41.2 μg/mL. This bioactivity was two times lower than the one demonstrated by the rest of farmed and wild *S. foetidus* counterparts, revealing a higher presence of cytotoxic compounds in its composition. By scrutinizing the % abundance and weight-normalized intensity values of metabolites among extracts, it is observed that indole-3-methylethanoate is present at significantly higher levels in the cytotoxic extract. Specifically, its abundance reaches 0.3% in the farmed extract #2, while it is detected at levels less than 0.1% in the rest of the tested samples. In terms of production levels, the weight-normalized intensity values of indole-3-methylethanoate differ by an order of magnitude between farmed extract #2 and the rest of analyzed *S. foetidus* samples. Although there is no report indicating the anticancer potential of this specific metabolite, indole compounds derived from marine sources, including sponges, have long been viewed to possess cytotoxic properties against tumor cell lines [141,142].

## 3. Materials and Methods

### 3.1. Sponge Material

Farmed individuals of *A. oroides* and *S. foetidus* were introduced in integrated aquaculture in June 2020, attached on cultivation structures (individual cages made from plastic mesh) in proximity (5–10 m distance) to the fish cages of an operating fish farm in Souda Bay, NW Crete, Aegean Sea (35.4801/24.1117), at 7–10 m depth. Farmed sponges originated from explants (i.e., biomass cuttings) collected from adjacent natural populations (Souda Bay, 35.4783/24.1091 for *A. oroides* and Stavros Bay, 35.5879/24.0785 for *S. foetidus*). For a continuous timespan of 19 months, the explants remained in open-sea cultivation, exhibiting negligible mortality and showing regeneration and positive growth (Vernadou et al., in preparation). Tissue sampling from three replicate individuals of each sponge species from wild and farmed sponge specimens (12 samples in total) were obtained in February 2022, according to Varamogianni-Mamatsi et al. (2022) [16]. Sampling locations for the wild individuals corresponded to the locations of collection for the initial seeding of the experimental sponge cultivation. Sampling was performed selectively by diving, and care was taken to partially collect excess biomass, thus, allowing the donor individuals to regenerate. In both cases (farmed and wild sponges), tissue samples were extracted underwater from a parent sponge, using a razor blade, and held in individual labeled sterile bags, prior to preservation in cooler boxes with ice packs and transportation to the facilities of Hellenic Centre for Marine Research within 3 h, where they were kept in −20 °C until further analysis.

The studied demosponge species are commonly distributed in high abundances along eastern Mediterranean habitats [143] with emerging bioremediation [16,24] and bioproduction potential [144] to be included in integrated aquaculture systems. The first sponge of interest, *Agelas oroides* (Schmidt, 1864), is a massive, variably lobate-digitate, vivid orange-colored demosponge that can reach 25 cm in height. It typically occurs in 2–40 m water depth, preferably in habitats with low light intensity [145,146]. Sponges of the genus *Agelas*, including the species *A. oroides*, are well-known alkaloid producers (e.g., pyrrole and terpenoid alkaloids) [49]. The second case study demosponge is the species *Sarcotragus foetidus* (Schmidt, 1862). This concerns a variably dark-colored, rather common Mediterranean keratose sponge, which approximates an irregularly globular to massive growth form, generally reaching 1 m in diameter and 50 cm in height. It is commonly found in shallow habitats exposed to light, but also in darker zones up to 400 m in depth [147]. This demosponge is known to host numerous symbiotic bacteria (e.g., heterotrophic bacteria and cyanobacteria [148]) and fungi [149]. *Sarcotragus* sponges are widely recognized to be prolific sources of a variety of bioactive compounds, such as terpenoids, indoles, as well as lipids [51].

### 3.2. Extraction

Freeze-dried specimens of wild and farmed sponges were ground in a conventional mixer to obtain a dry powder (1 to 4 g per specimen), which was subsequently extracted three times in an ice-cold sonication bath (15 min each round) using a solvent mixture of methanol/dichloromethane (20 mL of MeOH:DCM 1:1, *v*/*v* per gram of sponge). After each extraction, sponge suspensions were centrifuged (8000× *g*, 7 min, 20 °C), and collected supernatants were passed through a filter paper and evaporated to dryness using a centrifugal vacuum evaporator (EZ-2 Plus; Genevac, United Kingdom). The dry extracts were redissolved in 4 mL MeOH:DCM 1:1, transferred into 50 mL Falcon tubes, and mixed with 16 mL acetonitrile. After overnight protein precipitation at −20 °C, the samples were centrifuged (10,000× *g*, 10 min, 4 °C) and collected supernatants were evaporated to dryness. For the removal of neutral lipids, each sample was applied onto a glass column (8 mm i.d.) packed with 1.5 g of silica gel (silica gel 60, particle size: 0.060–0.200 mm, Merck; activated at 300 °C for 3 h) and elution was performed using 2% *v*/*v* ethyl acetate in hexane (20 mL), ethyl acetate (15 mL), and MeOH (15 mL). The latter two fractions were combined, evaporated to dryness, and stored at +4 °C until further analysis.

### 3.3. LC−MS/MS Analysis

#### 3.3.1. Chemicals and Reagents

Analytical solvents, MeOH, and acetonitrile solvents at Ultra-High-Performance Liquid Chromatography-Mass Spectrometry (UHPLC–MS) grade and formic acid at LC–MS grade were supplied from Carlo Erba^®^ Reagents S.A.S (Le Vaudreuil, France). Ultrapure water was supplied from a Milli-Q^®^ ultrapure water system equipped with a Milli-Q^®^ Reference and a Q-POD^®^ element.

#### 3.3.2. Instrumental LC–MS/MS Analysis

The LC–MS/MS analysis was performed using a Dionex^®^ Ultimate 3000 System (UHPLC, Thermo Scientific, Germany) coupled to a TSQ QuantisTM triple-stage quadrupole mass spectrometer (Thermo Scientific, Waltham, MA, USA). The UHPLC was equipped with four modules, a SR−3000 Solvent Rack, an LPG-3400RS pump, an WPS-3000TRS auto sampler with temperature control, and a TCC-3000RS column compartment. The triple-stage quadrupole mass spectrometer was equipped with an electrospray ionization (ESI) source. The LC−MS/MS operation and acquisition data system was controlled by the XCaliburTM 4.1 Thermo Scientific SP1 (0388-00CD-7B33, USA) software.

#### 3.3.3. Sample Preparation for LC–MS/MS Analysis

All plastic materials and glassware were cleaned carefully to avoid contamination. Organic solvents (LC−MS grade) and distilled water were analyzed before use, to minimize background interferences.

Before injection into the LC–MS/MS system, all extracts were dissolved in 1 mL MeOH, filtered with a 13-mm, 0.22-μm nylon syringe filter (Filter-Lab^®^, Sant Pere de Riudebitlles, Spain) using a 500 μL syringe (Gastight 1750 Hamilton^®^, Vernon Hills, IL, USA), and diluted 10 times with MeOH. The liquid extract was transferred using a syringe filter (Filter-Lab^®^, Sant Pere de Riudebitlles, Spain) into a conical insert into a sterile 2-mL vial (9-425 C0000752) with a screw cap and red PTFE/white silicone septa (Alwsci^®^ Technologies, Shaoxing, China).

#### 3.3.4. Chromatographic and Mass Spectrometry Conditions

The separation of compounds was achieved using an Accurore^TM^ RP-MS Column (2.6 μm, 150 × 2.1 mm, Thermo Fisher Scientific), by setting the sample injection volume to 10 μL. The gradient mobile phase consisted of water with 0.1% formic acid (A) and acetonitrile (B). Samples were injected and eluted at a flow of 0.200 mL/min with the following linear gradients: a 1 min re-equilibration phase at 5% B, 0.0–15.0 min at 5–50% of B, 15.0–20.0 min at 50–99% of B, 20.0–29.0 min at 99% of B, and 29.0–30.0 min at 99–5% of B.

Mass spectrometry (MS) analysis was carried out using the triple-stage quadrupole mass spectrometer. The electrospray ionization (ESI) parameters were set as follows: spray voltage, +3500/−3000 V; sheath gas flow, 50 L/min; auxiliary gas flow, 10 L min/L; sweep gas flow, 0 L min/L; ion transfer tube temperature, 320 °C; vaporizer temperature, 30 °C. The cycle time was set to 0.5 s, using a calibrated radio frequency (RF) lens and a collision induced dissociation (CID) gas of 1.5 mTorr. The collision energy was tested to 10, 20, and 40 V. Samples were injected in select reaction monitoring (SRM) mode, using multiple reaction monitoring (MRM). Selective MRM transitions were monitored for each targeted analyte, according to Tables S1 and S4 settings, and are provided, along with the instrumental parameter fragmentation settings used for the mass spectrometry conditions, as supplementary material. An intensive literature review was performed for each sponge’s species under study, on previously reported compounds that have been identified along with their mass ions, adducts and their fragments. Briefly, the identification and classification of detected metabolites was performed by reference to the literature and to public databases (e.g., DrugBank, FoodB, GNPS, HMDB, MoNA, Metabolomics Workbench, and PubChem).

### 3.4. Antibacterial Assays

The antibacterial activity of the crude sponge extracts was evaluated by performing growth inhibition assays for two strains belonging to the Gram-positive and -negative human opportunistic pathogenic bacteria. The tested strains were the methicillin-resistant *Staphylococcus aureus* strain COL (MRSA) and the *Escherichia coli* strain ATCC 25922, respectively. The protocol followed was based on the one proposed by Pinto-Almeida et al. (2022) [150]. In detail, *S. aureus* strains were cultured in tryptic soy broth (TSB; Becton Dickinson, Germany), and *E. coli* cells in Lysogeny broth (NZYtech), at 37 °C. The assays were performed in 96-well polystyrene flat bottom microplates. Bacterial overnight cultures were diluted to an optical density (OD_600nm_) of 0.005 and were incubated statically in the presence of different concentrations of each crude extract, solubilized in DMSO (1% *w*/*v*). All cultures were two-fold serially diluted, resulting in final concentrations of the extracts ranging from 250 to 0.4935 μg/mL. After 24 h of incubation at 37 °C, the minimal inhibitory concentration (MIC) value was determined by visual inspection. The latter is defined as the lowest concentration of an antimicrobial agent that inhibits the visible growth of a microorganism after overnight incubation [151]. The resulting values were compared with a positive control (vancomycin for MRSA, and tetracycline for *E. coli*), a DMSO solvent control, and a negative control (inoculated medium without any extract addition) on the same plate.

### 3.5. Anticancer Assays

The sponge crude extracts of wild and farmed populations of *A. oroides* and *S. foetidus* were tested in vitro against the human colorectal carcinoma cell line HCT-116 (ECACC 91091005, Porton Down, UK), according to Florindo et al. (2016) [152] and Prieto-Davó et al. (2016) [153]. Cells were cultured in McCoy’s 5A medium, supplemented with 10% FBS and 1% antibiotic/antimycotic solution (Gibco, Thermo Fisher Scientific, Paisley, UK) and maintained at 37 °C under a humidified atmosphere of 5% CO_2_. For cell viability assays, HCT-116 cells were seeded in 96-well plates (0.5 × 10^4^ cells/well). After 24 h, treatment with the marine sponges’ extracts (concentrations ranging from 0.30 to 125 µg/mL), DMSO (vehicle control), or 10 μM 5-fluorouracil (5-Fu, positive control) was followed for 72 h. Cell viability was assessed through MTS metabolism, using CellTiter 96^®^ Aqueous Non-Radioactive Cell Proliferation Assay (Promega, Fitchburg, WI, USA), following the manufacturer’s instructions. Absorbance signal (490 nm) was recorded using a Glomax^®^-MultiDetection System (Promega). All data were expressed as the mean ± standard error of the mean (SEM) from at least three independent experiments. Data analysis was performed using GraphPad Prism 8.4.2 software (La Jolla, CA, USA). Dose–response curves were established and IC_50_ best-fit values determined using the log-(inhibitor) vs. response–variable slope (four parameters).

### 3.6. Statistical Analysis

Principal component analysis (PCA) was performed using the XLSTAT software (version 2016; Addinsoft Inc., New York, NY, USA) to identify similarities/differences between the extracts obtained from wild and farmed sponge populations, with respect to their chemical composition. The content of each detected metabolite in the extracts was determined by using the peak intensity data generated from the LC–MS/MS analysis. Peak intensity percentages of constituents were used as active variables in PCA. Metabolites that gave zero-peak intensity values were excluded from the analysis. *t*-tests were performed using MetaboAnalyst 5.0. online platform (http://www.metaboanalyst.ca; accessed on 13 March 2023) to investigate significant differences in the content and production levels (expressed as weight-normalized intensity values) of the various metabolites present in both wild and farmed sponge populations. The same was applied to the IC_50_ values obtained from the different sponge populations, in order to compare their respective anticancer activities. The level of significance was set to p = 0.05.

## 4. Conclusions

Open-sea sponge farming is regarded as a promising source of high added-value natural products, in addition to effective cleanup technology. In this study, cultivations of the sponges *A. oroides* and *S. foetidus* were assessed for their metabolic profiling, as well as their bioactivities, and the results were compared with those obtained from their wild counterparts.

LC–MS/MS analysis revealed an array of natural products present in the studied extracts, with bioactive compounds belonging to alkaloids, benzenoids, indoles, lipids, polyketides, and other chemical classes. However, the biosynthesis of metabolites seemed to be species-specific, with alkaloids being the predominant constituents of *A. oroides* extracts, and lipids representing the major components for *S. foetidus*. In both cases, farming did not impose inhibition or alterations to the compositions and production levels of the sponge-related metabolites, while in some cases it even promoted the production of the bioactive compounds.

In terms of bioactivity, farmed sponge extracts had similar or slightly lower antimicrobial potency against Gram-positive strains than that demonstrated by their wild counterparts. A reverse effect was observed only in the case of *A. oroides* sponges, regarding Gram-negative bacteria. Extracts belonging to *S. foetidus* populations showed consistent, but marginal, anticancer activity against the human colon carcinoma cell line HCT-116, a bioactivity that was not detected in *A. oroides*.

Our findings demonstrate the significance of sponge mariculture in reproducing individuals of similar chemical fingerprints and bioactivities when compared with their wild donors. Although open-sea sponge farming can potentially serve as an additional source of profit for aquaculture-related enterprises, the “sponge-driven bioproduction/bioremediation” concept is still new and unexplored. Altogether, our study emphasizes the significance of sponge mariculture as a promising prospect for diversifying fish farm productivity through the growth of biotechnologically important marine invertebrates, with the possibility for future bioremediation applications and bioactive metabolites supply.

## Figures and Tables

**Table 1:** Distribution of metabolite superclasses, classes, and subclasses in the extracts of wild and farmed *Agelas oroides* sponges. Numbers in parentheses represent the standard deviation. ND—not detected.

Superclass	Class	Subclass	Wild (%)	Farmed (%)
Alkaloids	Pyrrole alkaloids	Linear pyrrole alkaloids	97.2 (1.4)	95.1 (0.9)
		Fused cyclic pyrrole alkaloids	0.9 (0.1)	0.7 (0.0)
		Dimeric pyrrole alkaloids	0.2 (0.2)	0.4 (0.1)
	Terpenoid alkaloids	ND	0.6 (0.5)	1.3 (0.1)
Indoles	ND	ND	<0.03	<0.03
Lipids	Fatty acyls	ND	1.0 (0.9)	2.4 (0.8)
	Glycerolipids	ND	<0.002	<0.003
	Steroids	ND	<0.002	<0.001

**Table 2:** Distribution of metabolite superclasses and classes in the extracts of wild and farmed *Sarcotragus foetidus* sponges. Numbers in parentheses represent the standard deviation. ND—not detected.

Superclass	Class	Wild (%)	Farmed (%)
Benzenoids	Anthracenes	<0.01	<0.01
	Benzene and substituted derivatives	4.2 (0.3)	3.2 (0.4)
	Benzopyrans	0.5 (0.2)	0.4 (0.0)
	Phenols	<0.01	<0.01
Dipeptides	ND	<0.02	<0.02
Indoles	ND	0.1 (0.0)	0.1 (0.1)
Lipids	Fatty acyls	55.5 (1.2)	56.2 (0.5)
	Glycerolipids	7.2 (0.4)	7.4 (0.3)
	Prenol lipids	1.5 (0.4)	3.1 (0.5)
	Steroids	30.1 (0.7)	28.7 (0.4)
Polyketides	ND	0.8 (0.1)	0.8 (0.0)

**Table 3:** Antimicrobial activity of *A. oroides* and *S. foetidus* crude extracts against methicillin-resistant *Staphylococcus aureus* (MRSA, strain COL) and *Escherichia coli* (strain ATCC 25922). MIC values are expressed in µg/mL. NA—not active at the tested concentrations. Vancomycin and tetracycline were used as positive controls for *S. aureus* and *E. coli* growth, respectively. Inoculated medium without any extract addition was used as negative control.

Species	Type of Sponge Population	Sponge Replicate	MIC Values (µg/mL) for *S. aureus* MRSA COL	MIC Values (µg/mL) for *E. coli* ATCC 25922
*Agelas oroides*	Wild	#1	NA	NA
		#2	NA	NA
		#3	250	NA
	Farmed	#1	NA	NA
		#2	250	NA
		#3	250	250
*Sarcotragus foetidus*	Wild	#1	125	NA
		#2	125	NA
		#3	250	NA
	Farmed	#1	NA	NA
		#2	250	NA
		#3	NA	NA
Positive control			1.9	3.9

**Table 4:** Marine sponge crude extracts with anticancer activity against human colorectal carcinoma cell line HCT-116. Results are expressed as mean IC_50_ values, determined by MTS assay. DMSO was used as vehicle control, and 10 µM 5-Fu as positive control.

Species	Type of Population	Replicate	IC_50_ (µg/mL)	95% CI
*Sarcotragus foetidus*	Wild	#1	70.8	70.3–80.4
		#2	70.4	65.7–74.7
		#3	71.5	68.4–73.8
	Farmed	#1	80.5	74.1–93.3
		#2	41.2	30.1–50.0
		#3	74.2	70.8–79.2

## Data Availability

The original data presented in the study are included in the article/Supplementary Material; further inquiries can be directed to the corresponding author.

## Supplementary Materials

The following supporting information can be downloaded at: https://www.mdpi.com/article/10.3390/md21120612/s1, Table S1: selected compounds for *A. oroides* extracts. Experimental retention time (RT), mode polarity (mode), collision energy, precursor ion, fragment ions, and bibliographic references for SRM ions. Table S2: the relative percentages of the metabolite components identified among the *A. oroides* extracts. Table S3: weight-normalized peak intensity values of the metabolite components identified among the *A. oroides* extracts, related to their production levels within sponges. Table S4: selected compounds for *S. foetidus* extracts. Experimental retention time (RT), mode polarity (mode), collision energy, precursor ion, fragment ions, and bibliographic references for SRM ions. Table S5: the relative percentages of the metabolite components identified among the *S. foetidus* extracts. Table S6: weight-normalized peak intensity values of the metabolite components identified among the *S. foetidus* extracts, related to their production levels within sponges [70,71,81,101,105,154,155,156,157].
