# Supplementary material for: Comparative Chemical Profiling and Antimicrobial/Anticancer Evaluation of Extracts from Farmed versus Wild Agelas oroides and Sarcotragus foetidus Sponges
Source: Mar Drugs. 2023 Nov 26;21(12):612. doi: 10.3390/md21120612 (PMC10744379; doi:10.3390/md21120612)
Supplement: Supplementary file 1 [file marinedrugs-21-00612-s001.zip › supplementary-2679535_preproof_final.pdf]

marine drugs

Supplementary Material

Comparative Chemical Profiling and Antimicrobial/Anticancer Evaluation of Extracts from Farmed versus Wild *Agelas oroides* and *Sarcotragus foetidus* Sponges

Despoina Varamogianni-Mamatsi [1,2,3,4,†], Maria João Nunes [5,†], Vanda Marques [6], Thekla I. Anastasiou [1], Eirini Kagiampaki [1], Emmanouela Vernadou [1], Thanos Dailianis [1], Nicolas Kalogerakis [2], Luís C. Branco [5], Cecília M. P. Rodrigues [6], Rita G. Sobral [3,4], Susana P. Gaudêncio [3,4,*] and Manolis Mandalakis [1,*]

[1] Institute of Marine Biology, Biotechnology and Aquaculture, Hellenic Centre for Marine Research, Heraklion Crete, 71500, Greece; d.varamogianni@hcmr.gr (D.V.-M.); theanast@hcmr.gr (T.I.A.); e.kagiampaki@hcmr.gr (E.K.); e.vernadou@hcmr.gr (E.V.); thanosd@hcmr.gr (T.D.)
[2] School of Chemical and Environmental Engineering, Technical University of Crete, Chania, 73100, Greece; nicolas.kalogerakis@enveng.tuc.gr (N.K.)
[3] Associate Laboratory i4HB, Institute for Health and Bioeconomy, NOVA School of Science and Technology, NOVA University of Lisbon, Campus Caparica, 2819-516 Caparica, Portugal; rgs@fct.unl.pt (R.G.S.)
[4] UCIBIO—Applied Molecular Biosciences Unit, Chemistry and Life Sciences Departments, NOVA School of Science and Technology, NOVA University of Lisbon, Campus Caparica, 2819-516 Caparica, Portugal
[5] LAQV, REQUIMTE, Associated Laboratory for Green Chemistry, Chemistry Department, NOVA School of Science and Technology, NOVA University of Lisbon, Campus Caparica, 2819-516 Caparica, Portugal; mjm.nunes@fct.unl.pt (M.J.N.); l.branco@fct.unl.pt (L.C.B.)
[6] Research Institute for Medicines (iMed.ULisboa), Faculty of Pharmacy, Universidade de Lisboa, Av. Professor Gama Pinto, 1649-003 Lisboa, Portugal; vismsmarques@ff.ulisboa.pt (V.M.); cmprodrigues@ff.ulisboa.pt (C.M.P.R.)

† The authors equality contributed to this work.

* Correspondence: mandalakis@hcmr.gr (M.M.); Tel.: +30-2810-337855 (M.M.); Fax: +30-2810-337822; s.gaudencio@fct.unl.pt (S.P.G.); Tel.: +351-212948300; Fax: +351-212948550

Table S1. Selected compounds for *A. oroides* extracts. Experimental retention time (RT), mode polarity (mode), collision energy, precursor ion, fragment ions and bibliographic references for SRM ions.

Superclass	Class	Chemical subclass	Metabolite	RT (min)	Mode	Collision Energy (V)	Chemical formula	Chemical structure	Precursor ion (m/z)	Fragments ions (m/z)	Ref for m/z
Alkaloids	Pyrrole alkaloids	Linear pyrrole alkaloids	Dispacamide A	12.52	+	20	$C_{11}H_{11}Br_2N_5O_2$	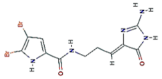	405.934	126.067; 138.068; 155.094	[71]
			Dispacamide B	1.58	+	10	$C_{11}H_{12}BrN_5O_2$	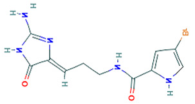	326.025	155.09; 326.00	[71]
			Hymenidin	11.35	+	10	$C_{11}H_{12}BrN_5O$	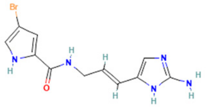	310.029	80.050; 110.071; 122.071	GNPS
			Keramadine	11.45	+	20	$C_{12}H_{15}BrN_5O^+$	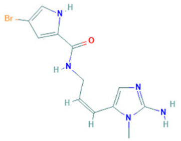	324.047	94.067; 136.088	[71]
			Oroidin	12.89	+	10	$C_{11}H_{11}Br_2N_5O$	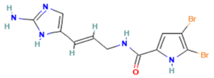	389.938	80.05; 110.07; 122.07; 139.10; 389.94	GNPS
			4,5-Dibromopyrrole-2-carboxylic acid	16.35	−	20	$C_5H_3Br_2NO_2$	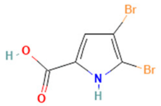	267.920	223.920	[154]

**Fused cyclic pyrrole alkaloids**	3-Debromohanishin	3.54	+	10	$C_{11}H_{14}BrN_2O_3^+$	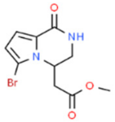	300.990	215.968; 254.978	[71]
	Dibromophakellin	12.88	+	40	$C_{11}H_{11}Br_2N_5O$	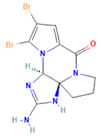	387.940	249.975; 328.892	GNPS
	Longamide B	14.31	+	20	$C_9H_8Br_2N_2O_3$	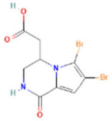	352.897	273.978; 292.876	[71]
	Longamide B methyl ester	16.56	+	20	$C_{10}H_{10}Br_2N_2O_3$	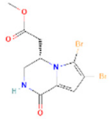	366.913	292.877	[71]
	Monobromoisophakellin	2.06	+	10	$C_{11}H_{12}BrN_5O$	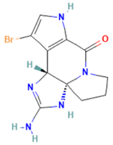	310.029	250.982	[70]
**Dimeric pyrrole alkaloids**	Ageliferin	Not Detected	+	10	$C_{22}H_{24}Br_2N_{10}O_2^+$	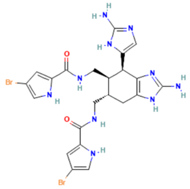	623.070	148.088; 160.888; 433.094; 450.120	[71]

	Bromoageliferin	13.73	+	10	$C_{22}H_{23}Br_3N_{10}O_2$	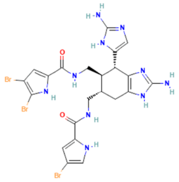	701.01	700.96	[71]
	Debromosceptrin acetate	26.33	+	20	$C_{22}H_{25}BrN_{10}O_2$	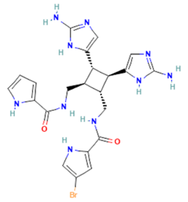	541.142	177.114	GNPS
	Nakamuric acid	26.21	+	20	$C_{20}H_{21}Br_2N_7O_4$	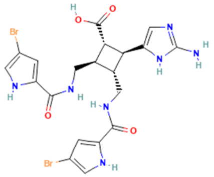	584.008	584.000	[155]
	Oxysceptrin	Not Detected	+	40	$C_{22}H_{25}Br_2N_{10}O_3^+$	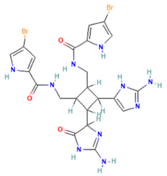	637.000	148.000; 177.000; 247.000; 466.00	[155]

	Sceptrin	18.14	+	20	$C_{22}H_{24}Br_2N_{10}O_2$	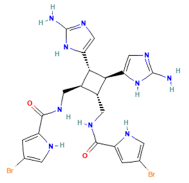	619.000	448.128; 243.131	GNPS
**Terpenoid alkaloids**	Agelasidine A	26.16	+	20	$C_{18}H_{33}N_3O_2S$	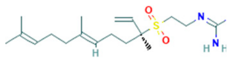	356.227	183.120; 184.140	[154]
	Agelasine	25.71	+	20	$C_{26}H_{40}N_5^+$	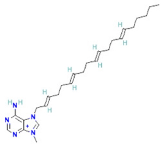	422.328	177.16	[156]
	Agelasine A	26.62	+	20	$C_{26}H_{40}ClN_5$	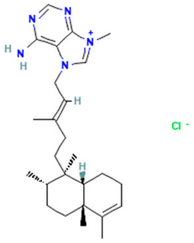	458.304	457.336	PubChem

		Agelasine E	Not Detected	+	40	$C_{26}H_{40}N_5^+$	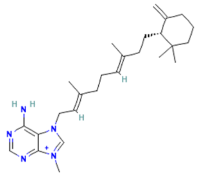	422.327	95.086; 121.01; 150.077; 423.331	GNPS
**Indoles**		4,6-Dihydroxyindole	2.82	+	10	$C_8H_7NO_2$	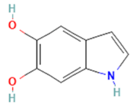	150.055	132.045; 133.029	FoodB
**Lipids**	**Fatty acyls**	10-Methyl-9(*Z*)-octadecenoic acid	26.18	–	40	$C_{19}H_{36}O_2$	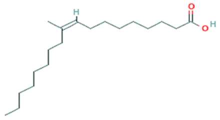	297.000	183.000	[81]
	**Glycerolipids**	2,3-Dihydroxypropyl(*Z*)-10-methyloctadec-9-enoate	25.37	+	10	$C_{22}H_{42}O_4$	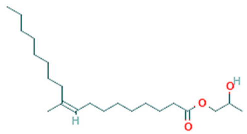	371.000	279.000; 297.000	[81]
	**Steroids**	20-Hydroxyecdysone-22-acetate	26.04	+	20	$C_{29}H_{46}O_8$	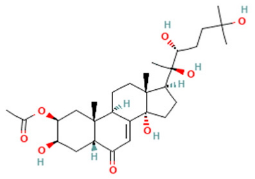	523.337	299.154; 531.331	GNPS

	β-ecdysterone	25.64	+	40	$C_{27}H_{44}O_7$	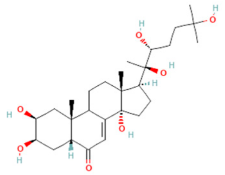	481.310	280.999; 299.154	GNPS
	Ponasterone-A	Not Detected	−	10	$C_{27}H_{44}O_6$	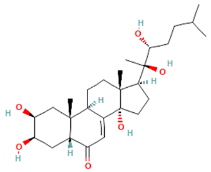	464.000	463.000; 928.000	MoNA
**Polyketides**	Trichodermanone C	Not Detected	−	40	$C_{20}H_{26}O_8$	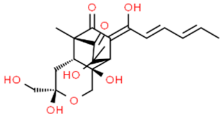	393.115	205.000; 247.000	[157]

Table S2. The relative percentages of the metabolite components identified among the *A. oroides* extracts. −; Not Detected.

	%Content					
**Metabolite**	**Wild #1**	**Wild #2**	**Wild #3**	**Farmed #1**	**Farmed #2**	**Farmed #3**
**Dispacamide A**	2.7%	1.1%	2.4%	1.5%	1.4%	1.8%
**Dispacamide B**	1.2%	5.5%	2.1%	8.3%	6.3%	8.9%
**Hymenidin**	2.6%	0.9%	0.7%	0.6%	0.6%	1.7%
**Keramadine**	0.5%	5.9%	4.5%	7.2%	6.8%	10.0%
**Oroidin**	82.8%	75.8%	82.7%	70.8%	73.0%	66.9%
**4,5-Dibromopyrrole-2-carboxylic acid**	6.9%	7.2%	6.4%	7.6%	6.1%	6.0%
**3-Debromohanishin**	<0.01%	<0.01%	<0.01%	<0.01%	<0.01%	<0.01%
**Dibromophakellin**	0.7%	0.7%	0.8%	0.6%	0.6%	0.6%
**Longamide B**	0.02%	0.02%	0.05%	0.01%	0.01%	0.01%
**Longamide B methyl ester**	0.1%	0.1%	0.1%	0.1%	0.1%	0.1%
**Monobromoisophakellin**	0.01%	0.01%	<0.01%	0.01%	0.01%	0.01%

**Ageliferin**	<0.01%	–	–	–	–	–
**Bromoageliferin**	0.01%	0.02%	<0.01%	0.01%	0.01%	0.01%
**Debromosceptrin acetate**	0.1%	0.1%	0.01%	0.1%	0.1%	0.1%
**Nakamuric acid**	0.3%	0.1%	0.01%	0.2%	0.3%	0.2%
**Oxysceptrin**	<0.01%	–	–	–	–	–
**Sceptrin**	<0.01%	<0.01%	<0.01%	<0.01%	<0.01%	–
**Agelasidine A**	0.1%	0.1%	0.01%	0.1%	0.1%	0.1%
**Agelasine**	0.1%	0.1%	0.01%	0.1%	0.1%	0.1%
**Agelasine A**	0.8%	0.7%	0.1%	1.1%	1.2%	0.9%
**Agelasine E**	<0.01%	–	<0.01%	–	<0.01%	–
**4,6-Dihydroxyindole**	0.01%	0.1%	<0.01%	0.01%	0.01%	0.1%
**10-Methyl-9(*Z*)-octadecenoic acid**	1.1%	1.9%	0.1%	1.7%	3.2%	2.4%
**2,3-Dihydroxypropyl(*Z*)-10-methyloctadec-9-enoate**	<0.01%	<0.01%	<0.01%	<0.01%	<0.01%	0.01%
**20-Hydroxyecdysone-22-acetate**	<0.01%	<0.01%	<0.01%	<0.01%	0.01%	<0.01%

**β-ecdysterone**	0.01%	0.01%	<0.01%	0.01%	0.01%	<0.01%
**Ponasterone-A**	0.01%	–	–	–	<0.01%	–
**Trichodermanone C**	<0.01%	–	–	–	–	–

Table S3. Weight-normalized peak intensity values of the metabolite components identified among the *A. oroides* extracts, related to their production levels within sponges. −; Not Detected.

	Weight-normalized Intensity (Intensity g_{sponge}^{-1})					
**Metabolite**	**Wild #1**	**Wild #2**	**Wild #3**	**Farmed #1**	**Farmed #2**	**Farmed #3**
**Dispacamide A**	2.7E+10	10.0E+10	2.1E+11	1.8E+10	2.0E+10	2.4E+10
**Dispacamide B**	1.2E+10	5.2E+10	1.8E+11	9.6E+10	9.3E+10	1.2E+11
**Hymenidin**	2.6E+10	8.1E+09	6.4E+10	7.4E+09	9.0E+09	2.2E+10
**Keramadine**	4.5E+09	5.6E+10	4.0E+11	8.3E+10	1.0E+11	1.3E+11
**Oroidin**	8.2E+11	7.2E+11	7.3E+12	8.2E+11	1.1E+12	8.9E+11
**4,5-Dibromopyrrole-2-carboxylic acid**	6.8E+10	6.8E+10	5.6E+11	8.9E+10	9.0E+10	8.0E+10
**3-Debromohanishin**	3.1E+07	7.0E+07	2.3E+07	2.3E+07	1.2E+08	3.1E+07
**Dibromophakellin**	7.2E+09	6.6E+09	7.0E+10	7.4E+09	9.4E+09	7.9E+09
**Longamide B**	2.1E+08	2.3E+08	4.5E+09	1.3E+08	2.2E+08	9.8E+07
**Longamide B methyl ester**	1.1E+09	8.3E+08	1.1E+10	1.1E+09	1.1E+09	8.1E+08
**Monobromoisophakellin**	1.3E+08	8.0E+07	2.8E+07	1.3E+08	8.3E+07	1.2E+08
**Ageliferin**	4.9E+06	–	–	–	–	–
**Bromoageliferin**	8.2E+07	1.5E+08	4.8E+07	1.3E+08	8.0E+07	8.8E+07
**Debromosceptrin acetate**	9.6E+08	7.5E+08	7.4E+08	7.4E+08	2.1E+09	1.8E+09
**Nakamuric acid**	2.6E+09	10.0E+08	9.5E+08	2.7E+09	4.7E+09	3.2E+09
**Oxysceptrin**	1.0E+07	–	–	–	–	–

**Sceptrin**	9.3E+06	6.7E+06	2.6E+07	3.6E+06	1.6E+07	–
**Agelasidine A**	9.6E+08	4.3E+08	4.8E+08	9.3E+08	1.8E+09	1.9E+09
**Agelasine**	1.2E+09	7.7E+08	6.5E+08	1.6E+09	1.9E+09	1.8E+09
**Agelasine A**	7.5E+09	7.0E+09	4.2E+09	1.2E+10	1.8E+10	1.2E+10
**Agelasine E**	8.2E+06	–	2.6E+07	–	9.2E+06	–
**4,6-Dihydroxyindole**	1.4E+08	4.8E+08	9.0E+07	9.0E+07	1.5E+08	8.7E+08
**10-Methyl-9(*Z*)-octadecenoic acid**	1.1E+10	1.8E+10	1.1E+10	1.9E+10	4.7E+10	3.2E+10
**2,3-Dihydroxypropyl(*Z*)-10-methyloctadec-9-enoate**	2.6E+07	1.8E+07	1.9E+07	3.5E+06	3.3E+07	7.5E+07
**20-Hydroxyecdysone-22-acetate**	4.8E+07	4.1E+07	2.2E+07	4.9E+07	8.7E+07	3.9E+07
**β-ecdysterone**	1.1E+08	6.7E+07	1.1E+07	5.9E+07	2.0E+08	3.0E+07
**Ponasterone-A**	5.8E+07	–	–	–	2.7E+07	–
**Trichodermanone C**	3.4E+06	–	–	–	–	–

Table S4. Selected compounds for *S. foetidus* extracts. Experimental retention time (RT), mode polarity (mode), collision energy, precursor ion, fragment ions and bibliographic references for SRM ions.

Superclass	Chemical class	Metabolite	RT (min)	Mode	Collision Energy (V)	Chemical formula	Chemical structure	Precursor ion (m/z)	Fragment ions (m/z)	Ref for m/z
Benzenoids	Anthracenes	Emodin	4.23	+	10	$C_{15}H_{10}O_5$	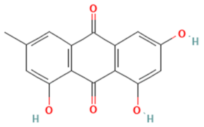	271.060	139.054; 225.055	GNPS
		Endocrocin	19.51	–	10	$C_{16}H_{10}O_7$	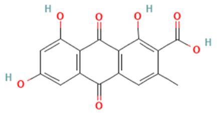	313.035	269.046; 313.037	GNPS
	Benzene & substituted derivatives	3-Phenylpropane-1,2-diol	1.54	+	10	$C_9H_{12}O_2$	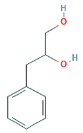	153.092	91.055; 93.070; 135.081; 153.092	DrugBank
		3,4-Dimethoxybenzoic acid	1.52	+	20	$C_9H_{10}O_4$	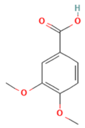	183.180	126.046; 152.062	GNPS
		8-*O*-4'-Dehydrodiferulic acid	20.53	+	10	$C_{20}H_{18}O_8$	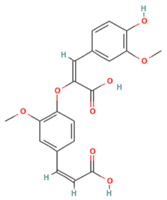	387.108	195.066; 351.087	HMDB

	Toluate	1.50	+	10	$C_8H_8O_2$	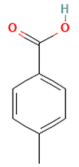	137.060	91.0555; 109.0661; 137.0597	[101]
Benzopyrans	3-Isochromanone	1.50	+	10	$C_9H_8O_2$	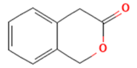	149.061	105.035; 121.028	[101]
	7-Hydroxy-2-(2-hydroxypropyl)-5-methylchromone (Aloesol)	1.51	+	10	$C_{13}H_{14}O_4$	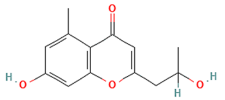	235.097	191.071; 217.087; 235.070	HMDB
Phenols	4-Hydroxyphenylacetic acid	4.46	+	20	$C_8H_8O_3$	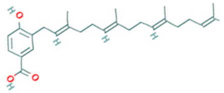	153.055	95.049; 107.049	GNPS
	Tyrosol	1.91	+	20	$C_8H_{10}O_2$	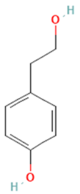	121.064	71.970	GNPS

**Dipeptides**		3-Nitropropionic acid	1.39	+	10	$C_3H_5NO_4$	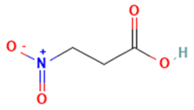	120.029	91.054; 100.001; 100.505	GNPS
**Indoles**		Indole-3-methylethanoate	1.49	+	20	$C_{11}H_{11}NO_2$	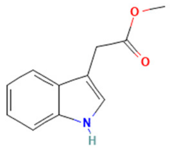	190.087	103.055; 128.049; 130.065	PubChem
**Lipids**	**Fatty acyls**	*N*-hexadecanoyl-*L*-homoserine lactone	12.05	+	10	$C_{20}H_{37}NO_3$	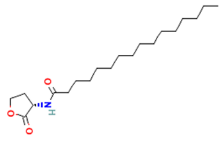	340.283	298.275; 322.274	[101]
	**Glycerolipids**	1-*O*-(2,3,4,5-tetrahydroxycyclopentyl)-3-*O*-hexadecylglycerol	27.44	+	10	$C_{24}H_{48}O_7$	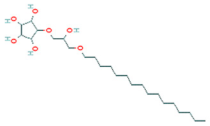	471.329	471.000	[105]
		1-*O*-(2,3,4,5-tetrahydroxycyclopentyl)-3-*O*-(10-methylhexadecyl) glycerol	28.23	+	10	$C_{25}H_{50}O_7$	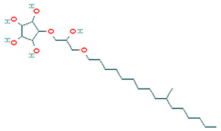	485.343	245.000; 485.000	[105]

	Monovaccenin	26.24	+	10	$C_{21}H_{40}O_4$	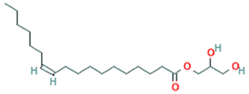	357.000	321.279; 339.282; 357.300	HMDB
	7E,12*E*,20*Z*-Variabilin	25.61	+	20	$C_{25}H_{34}O_4$	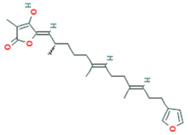	399.254	107.083; 135.078	GNPS
	4-Hydroxy-3-tetraprenylbenzoic acid	27.75	+	10	$C_{27}H_{38}O_3$	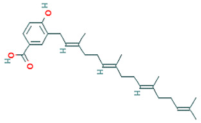	411.287	151.038; 411.287	GNPS
**Prenol lipids**	1,4-Dihydroxy-2-tetraprenylbenzene	28.67	+	10	$C_{26}H_{38}O_2$	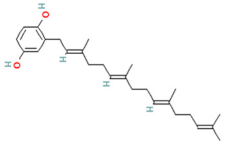	383.292	109.1007; 383.2932	GNPS
	(+)-12,15 Dihydroxycurcuphenol	23.12	+	20	$C_{15}H_{22}O_3$	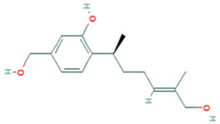	251.164	59.050; 149.025	[101]

	24-Methylcholest-7-en-3β-ol	22.68	+	40	$C_{28}H_{48}O$	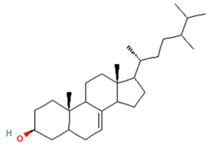	400.371	85.102; 297.852	FoodB
	24-Methylcholesta-5,7-dien-3β-ol (22,23-Dihydroergosterol)	12.52	+	10	$C_{28}H_{46}O$	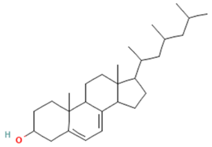	398.355	381.352; 399.363	HMDB
**Steroids**	24-Methylcholesta-7,22-dien-3β-ol (Stellasterol)	11.58	+	10	$C_{28}H_{46}O$	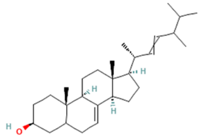	398.355	295.243; 399.363	FoodB
	24-Methylcholesta-5,7,22-trien-3β-ol (Ergosterol)	12.51	+	10	$C_{28}H_{44}O$	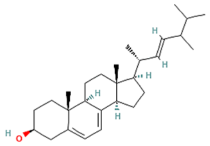	397.35	204.118; 397.116	GNPS
	Cholest-7-en-3β-yl acetate	27.15	+	10	$C_{29}H_{48}O_2$	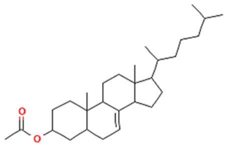	429.372	159.115; 411.362	GNPS

	Cholesta-5,7-dien-3β-ol (Δ^7-Cholesterol)	27.85	+	10	$C_{27}H_{44}O$	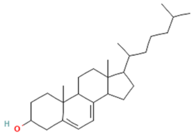	385.346	161.1325; 173.1326; 367.3349	GNPS
**Polyketides**	3,8-Dihydroxy-6-methoxy-8-methylxanthone (Griseoxanthone C)	23.17	+	10	$C_{15}H_{12}O_5$	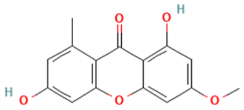	273.075	230.0581; 273.0766	GNPS
	Chrysophanol	1.53	+	10	$C_{15}H_{10}O_4$	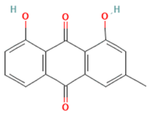	255.066	209.060; 255.065	GNPS
	Dechlorogriseofulvin	14.99	+	20	$C_{17}H_{18}O_6$	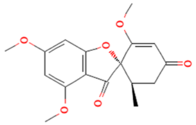	319.118	165.054	GNPS
	Griseofulvin	1.53	+	10	$C_{17}H_{17}ClO_6$	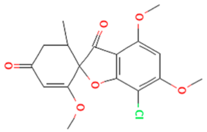	353.079	385.079; 353.079	GNPS
	Norlichexanthone	21.03	−	10	$C_{14}H_{10}O_5$	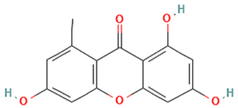	257.045	257.108; 257.304; 257.0455	GNPS

Table S5. The relative percentages of the metabolite components identified among the *S. foetidus* extracts. −; Not Detected.

	%Content					
**Metabolite**	**Wild #1**	**Wild #2**	**Wild #3**	**Farmed #1**	**Farmed #2**	**Farmed #3**
**Emodin**	<0.01%	<0.01%	<0.01%	–	<0.01%	–
**Endocrocin**	<0.01%	<0.01%	0.01%	<0.01%	<0.01%	<0.01%
**3-Phenylpropane-1,2-diol**	0.4%	0.4%	0.5%	0.2%	0.3%	0.4%
**3,4-Dimethoxybenzoic acid**	0.1%	0.1%	0.1%	0.1%	0.1%	0.1%
**8-*O*-4'-Dehydrodiferulic acid**	1.2%	1.8%	1.8%	1.3%	1.1%	1.5%
**Toluate**	2.8%	2.1%	1.5%	1.8%	1.8%	1.5%
**3-Isochromanone**	0.1%	0.1%	0.2%	0.1%	0.1%	0.1%

**7-Hydroxy-2-(2-hydroxypropyl)-5-methylchromone**	0.4%	0.2%	0.5%	0.4%	0.3%	0.3%
**4-Hydroxyphenylacetic acid**	0.01%	<0.01%	0.01%	<0.01%	<0.01%	<0.01%
**Tyrosol**	0.01%	0.01%	<0.01%	0.01%	<0.01%	0.01%
**3-Nitropropionic acid**	0.01%	0.01%	0.02%	0.02%	0.01%	0.01%
**Indole-3-methylethanoate**	0.1%	0.1%	0.1%	0.1%	0.3%	0.1%
***N*-hexadecanoyl-*L*-homoserine lactone**	56.2%	54.1%	56.3%	55.9%	56.7%	56.0%
**1-*O*-(2,3,4,5-tetrahydroxycyclopentyl)-3-*O*-(10-methylhexadecyl) glycerol**	2.3%	2.4%	2.6%	2.6%	2.3%	2.5%
**1-*O*-(2,3,4,5-tetrahydroxycyclopentyl)-3-*O*-hexadecylglycerol**	0.2%	0.3%	0.2%	0.2%	0.3%	0.4%
**Monovaccenin**	4.2%	4.9%	4.4%	4.9%	4.7%	4.5%
**7*E*,12*E*,20*Z*-Variabilin**	0.03%	0.01%	0.01%	0.01%	0.01%	0.01%

**4-Hydroxy-3-tetraprenylbenzoic acid**	1.1%	1.8%	0.9%	2.2%	2.7%	3.0%
**1,4-Dihydroxy-2-tetraprenylbenzene**	0.04%	0.2%	0.4%	0.4%	0.4%	0.5%
**(+)-12,15 Dihydroxycurcuphenol**	<0.01%	0.01%	<0.01%	0.03%	0.06%	0.05%
**24-Methylcholest-7-en-3β-ol**	0.07%	0.05%	0.08%	0.05%	0.08%	0.05%
**24-Methylcholesta-5,7-dien-3β-ol**	0.01%	0.02%	0.02%	0.01%	0.02%	0.03%
**24-Methylcholesta-7,22-dien-3β-ol**	0.01%	0.01%	0.01%	0.01%	0.01%	0.01%
**24-Methylcholesta-5,7,22-trien-3β-ol (Ergosterol)**	29.9%	30.8%	29.3%	29.0%	28.4%	28.3%
**Cholest-7-en-3β-yl acetate**	–	<0.01%	<0.01%	<0.01%	<0.01%	<0.01%
**Cholesta-5,7-dien-3β-ol**	0.05%	0.07%	0.05%	0.06%	0.06%	0.06%
**3,8-Dihydroxy-6-methoxy-8-methylxanthone**	0.3%	0.3%	0.3%	0.3%	0.3%	0.3%

**Chrysophanol**	0.4%	0.3%	0.3%	0.2%	0.3%	0.2%
**Dechlorogriseofulvin**	<0.01%	<0.01%	<0.01%	<0.01%	<0.01%	–
**Griseofulvin**	0.2%	0.1%	0.2%	0.1%	0.1%	0.1%
**Norlichexanthone**	0.1%	0.1%	0.1%	0.1%	0.1%	0.1%

Table S6. Weight-normalized peak intensity values of the metabolite components identified among the *S. foetidus* extracts, related to their production levels within sponges. −; Not Detected.

	Weight-normalized Intensity (Intensity g_{sponge}^{-1})					
**Metabolite**	**Wild #1**	**Wild #2**	**Wild #3**	**Farmed #1**	**Farmed #2**	**Farmed #3**
**Emodin**	1.7E+07	7.3E+07	2.2E+08	−	2.0E+07	−
**Endocrocin**	3.3E+07	2.4E+08	4.2E+08	5.0E+07	4.4E+07	3.0E+07
**3-Phenylpropane-1,2-diol**	1.9E+10	1.9E+10	2.3E+10	1.1E+10	1.1E+10	2.4E+10
**3,4-Dimethoxybenzoic acid**	3.1E+09	2.9E+09	4.4E+09	3.2E+09	2.3E+09	3.1E+09
**8-*O*-4'-Dehydrodiferulic acid**	6.0E+10	9.7E+10	9.0E+10	7.0E+10	4.5E+10	9.7E+10
**Toluate**	1.4E+11	1.1E+11	7.8E+10	9.4E+10	6.0E+10	9.6E+10
**3-Isochromanone**	6.5E+09	6.2E+09	1.1E+10	6.2E+09	5.9E+09	8.5E+09

**7-Hydroxy-2-(2-hydroxypropyl)-5-methylchromone**	2.0E+10	1.3E+10	2.7E+10	1.9E+10	1.2E+10	1.7E+10
**4-Hydroxyphenylacetic acid**	2.7E+08	8.1E+07	2.6E+08	1.4E+08	1.1E+08	1.4E+08
**Tyrosol**	3.9E+08	3.5E+08	1.5E+08	3.0+08	1.0E+08	3.5E+08
**3-Nitropropionic acid**	7.3E+08	7.0E+08	1.2E+09	1.0E+09	6.4E+08	5.8E+08
**Indole-3-methylethanoate**	5.0E+09	5.4E+09	6.6E+09	3.7E+09	1.2E+10	5.8E+09
***N*-hexadecanoyl-*L*-homoserine lactone**	2.8E+12	3.0E+12	2.8E+12	3.0E+12	2.4E+12	3.7E+12
**1-*O*-(2,3,4,5-tetrahydroxycyclopentyl)-3-*O*-(10-methylhexadecyl)glycerol**	1.2E+11	1.3E+11	1.3E+11	1.4E+11	1.0E+11	1.6E+11
**1-*O*-(2,3,4,5-tetrahydroxycyclopentyl)-3-*O*-hexadecylglycerol**	9.6E+09	1.6E+10	1.1E+10	1.1E+10	1.1E+10	2.4E+10
**Monovaccenin**	2.1E+11	2.7E+11	2.2E+11	2.6E+11	2.0E+11	2.9E+11
**7*E*,12*E*,20*Z*-Variabilin**	1.6E+09	6.4E+08	5.1E+08	3.0E+08	6.0E+08	9.5E+08

**4-Hydroxy-3-tetraprenylbenzoic acid**	5.6E+10	9.6E+10	4.5E+10	1.2E+11	1.1E+11	2.0E+11
**1,4-Dihydroxy-2-tetraprenylbenzene**	1.9E+09	10.0E+10	1.9E+10	2.1E+10	1.9E+10	3.3E+10
**(+)-12,15 Dihydroxycurcuphenol**	3.2E+07	3.3E+08	4.3E+07	1.7E+09	2.6E+09	3.1E+09
**24-Methylcholest-7-en-3β-ol**	3.5E+09	2.6E+09	3.9E+09	2.5E+09	3.3E+09	3.1E+09
**24-Methylcholesta-5,7-dien-3β-ol**	5.6E+08	1.3E+09	1.1E+09	6.1E+08	8.7E+08	2.0E+09
**24-Methylcholesta-7,22-dien-3β-ol**	6.4E+08	3.5E+08	6.5E+08	4.1E+08	4.1E+08	3.7E+08
**24-Methylcholesta-5,7,22-trien-3β-ol (Ergosterol)**	1.5E+12	1.7E+12	1.5E+12	1.6E+12	1.2E+12	1.9E+12
**Cholest-7-en-3β-yl acetate**	–	4.7E+07	1.4E+08	1.7E+08	9.5E+07	1.6E+08
**Cholesta-5,7-dien-3β-ol**	2.6E+09	3.6E+09	2.5E+09	3.4E+09	2.5E+09	4.0E+09
**3,8-Dihydroxy-6-methoxy-8-methylxanthone**	1.3E+10	1.6E+10	1.6E+10	1.7E+10	1.3E+10	1.8E+10

**Chrysophanol**	1.7E+10	1.4E+10	1.6E+10	1.1E+10	1.2E+10	1.6E+10
**Dechlorogriseofulvin**	1.5E+07	1.5E+07	2.2E+07	2.1E+07	6.5E+07	–
**Griseofulvin**	8.2E+09	5.4E+09	8.4E+09	7.1E+09	5.8E+09	9.1E+09
**Norlichexanthone**	4.0E+09	4.8E+09	4.1E+09	4.9E+09	4.1E+09	6.3E+09
